# Sperm acrosome overgrowth and infertility in mice lacking chromosome 18 pachytene piRNA

**DOI:** 10.1371/journal.pgen.1009485

**Published:** 2021-04-08

**Authors:** Heejin Choi, Zhengpin Wang, Jurrien Dean

**Affiliations:** Laboratory of Cellular and Developmental Biology, NIDDK, National Institutes of Health, Bethesda, MD, United States of America; Cornell University, UNITED STATES

## Abstract

piRNAs are small non-coding RNAs required to maintain genome integrity and preserve RNA homeostasis during male gametogenesis. In murine adult testes, the highest levels of piRNAs are present in the pachytene stage of meiosis, but their mode of action and function remain incompletely understood. We previously reported that BTBD18 binds to 50 pachytene piRNA-producing loci. Here we show that spermatozoa in gene-edited mice lacking a BTBD18 targeted pachytene piRNA cluster on Chr18 have severe sperm head dysmorphology, poor motility, impaired acrosome exocytosis, zona pellucida penetration and are sterile. The mutant phenotype arises from aberrant formation of proacrosomal vesicles, distortion of the *trans*-Golgi network, and up-regulation of GOLGA2 transcripts and protein associated with acrosome dysgenesis. Collectively, our findings reveal central role of pachytene piRNAs in controlling spermiogenesis and male fertility.

## Author summary

piRNAs are major non-coding small RNAs in the male gonad that are essential for male germ cell development. In adult mouse testis, the most highly enriched piRNAs are expressed at the pachytene stage of meiosis but the function and mode of action are largely unknown. To investigate the role of pachytene piRNAs during spermatogenesis, we have used CRISPR/Cas9 to establish a mouse line with ~1.3 kb deletion in the promoter of bi-directional piRNA precursor locus on chromosome 18 (*pi18*). Although mature spermatozoa are observed, the male mice are sterile due to defects in sperm motility, head morphology, acrosome exocytosis, and interactions with female gametes. Using electron microscopy and RNA-seq, we confirm the role of *pi18* pachytene piRNAs in regulating Golga2 transcripts during acrosome biogenesis resulting in a severe overgrowth phenotype. This study identifies essential roles of *pi18* pachytene piRNAs in controlling and orchestrating transcriptional programs by regulating spermatogenic mRNAs.

## Introduction

Genome integrity and RNA homeostasis are essential for mammalian gametogenesis and rely on miRNA (microRNAs), siRNAs (small interfering RNAs) and piRNAs (P-element induced wimpy testis [PIWI]-interacting RNAs) [1,2]. piRNAs are the most abundant population of small, non-coding RNAs in male gonads. During mouse spermatogenesis, germ cells produce pre-pachytene piRNAs derived from transposable elements and subsequently generate pachytene piRNAs from distinctive loci scattered throughout the genome [3–5]. The most well-studied and conserved function of pre-pachytene piRNAs is repression of transposons to ensure integrity of the germline genome [6–12]. Pachytene piRNAs derive their designation from expression during meiosis and are considerably more abundant than pre-pachytene piRNAs [13]. Although mice lacking proteins required for pachytene piRNA biogenesis have spermatogenic arrest and male sterility [5,14–17], the functions of pachytene piRNAs themselves are much less understood. Hypotheses for their role include: 1) cleaving mRNAs necessary for meiotic progression; and 2) directed degradation of target mRNA analogous to miRNA function in somatic cells have been proposed [18–21]. However, despite being the major mouse piRNA cluster, 17-qA3.3-27363(-),26735(+) inactivation has no discernable phenotype or impact on male fertility [22]. This suggests either extensive genetic redundancy among pachytene piRNAs or a lack of biological function. A recent study provides the first evidence that deletion of the promoter of the bi-directionally transcribed pachytene piRNA cluster 6-qF3-28913(-),8009(+) leads to severe male subfertility [23]. Thus, pachytene piRNAs play essential roles in murine male fertility.

Mouse spermatogenesis has three distinct phases: 1) mitotic proliferation and differentiation; 2) meiosis with two reductive divisions to form haploid gametes; and 3) spermiogenesis in which terminally differentiated, round spermatids undergo a remarkable transformation. During this latter process, male germ cells shed cytoplasmic droplets and transmogrify into elongated mature spermatozoa with a sperm-unique acrosome overlying a condensed nucleus, a mid-piece filled with mitochondria and a flagellum for forward motility necessary to pass through the female reproductive tract and fertilize eggs. The 16 stages of spermiogenesis are divided into four phases: Golgi (stages 1–3), cap (stages 4–7), acrosome (stages 8–12), and maturation (stages 13–16) [24–28]. The acrosome is a specialized subcellular, membranous organelle located at the anterior portion of the sperm head. It is an exocytotic vesicle that contains enzymes essential for fertilization, dispersion of cumulus cells and/or sperm penetration of the zona pellucida [24,25,29,30]. Acrosome biogenesis begins in the concave region of the spermatid nucleus during the Golgi phase of spermiogenesis. Golgi-derived proacrosomal vesicles (PVs) accumulate and a single large acrosomal granule (AG) is formed by fusion of small vesicles. The AG attaches to the nuclear envelope via the acroplaxome (Apx), a structure that lies between the inner membrane of the acrosome and nucleus [26]. By combining with additional Golgi-derived vesicles, the size of the acrosome increases and spreads over the anterior nuclear pole during the cap phase of spermiogenesis. The subsequent elongation phase which forms mature spermatozoa is mediated by the perinuclear ring of the manchette and its associated microtubules that are subsequently degraded. The manchette is a temporary microtubular/actin-containing structure that is critical for acrosomal vesicle formation, macromolecule transport to the centrosome and development of the spermatid principal piece (tail) [27]. Despite the well-documented morphologic changes in acrosome biogenesis during spermiogenesis, the underlying molecular mechanisms remain to be determined. Because of chromatin condensation in which nuclear histones are replaced with disulfide-bond rich protamines, elongating spermatids and mature sperm are transcriptionally silent. Thus, accurate post-transcriptional quality control of RNA and proteins is critical for normal spermatogenesis.

We previously identified a pachytene germ cell nuclear protein, BTBD18, that acts as a licensing factor for RNA polymerase II elongation at fifty pachytene piRNA sites scattered across autosomes. About half of these sites are transcribed on alternative strands of DNA from bi-directional, A-MYB and BTBD18-binding promoters. The absence of BTBD18 in *Btbd18*^*Null*^ mice disrupts piRNA biogenesis, arrests spermiogenesis at an early stage and results in male sterility [31]. To explore the functional importance of pachytene piRNAs, we have used CRISPR/Cas9 genome editing to establish mouse lines unable to express the bi-directional pachytene piRNA cluster 18-qE-36451.1(-),1295(+) (referred to as *pi18*). Although mice lacking *pi18* pachytene piRNAs produce mature spermatozoa, the mutant sperm have strikingly overgrown acrosomes with severely reduced hyperactivity rendering them unable to penetrate the zona pellucida surrounding eggs and are sterile. By investigating the transcriptome profiles of *pi18* mutant testicular germ cells, we discovered increased abundance of *Golga2* transcripts associated with acrosome overgrowth. Taken together our data indicate that *pi18* pachytene piRNAs play an essential role during spermiogenesis which is critical for male fertility.

## Results

### *pi18* pachytene piRNAs are required for spermatogenesis

**Fig 1:**
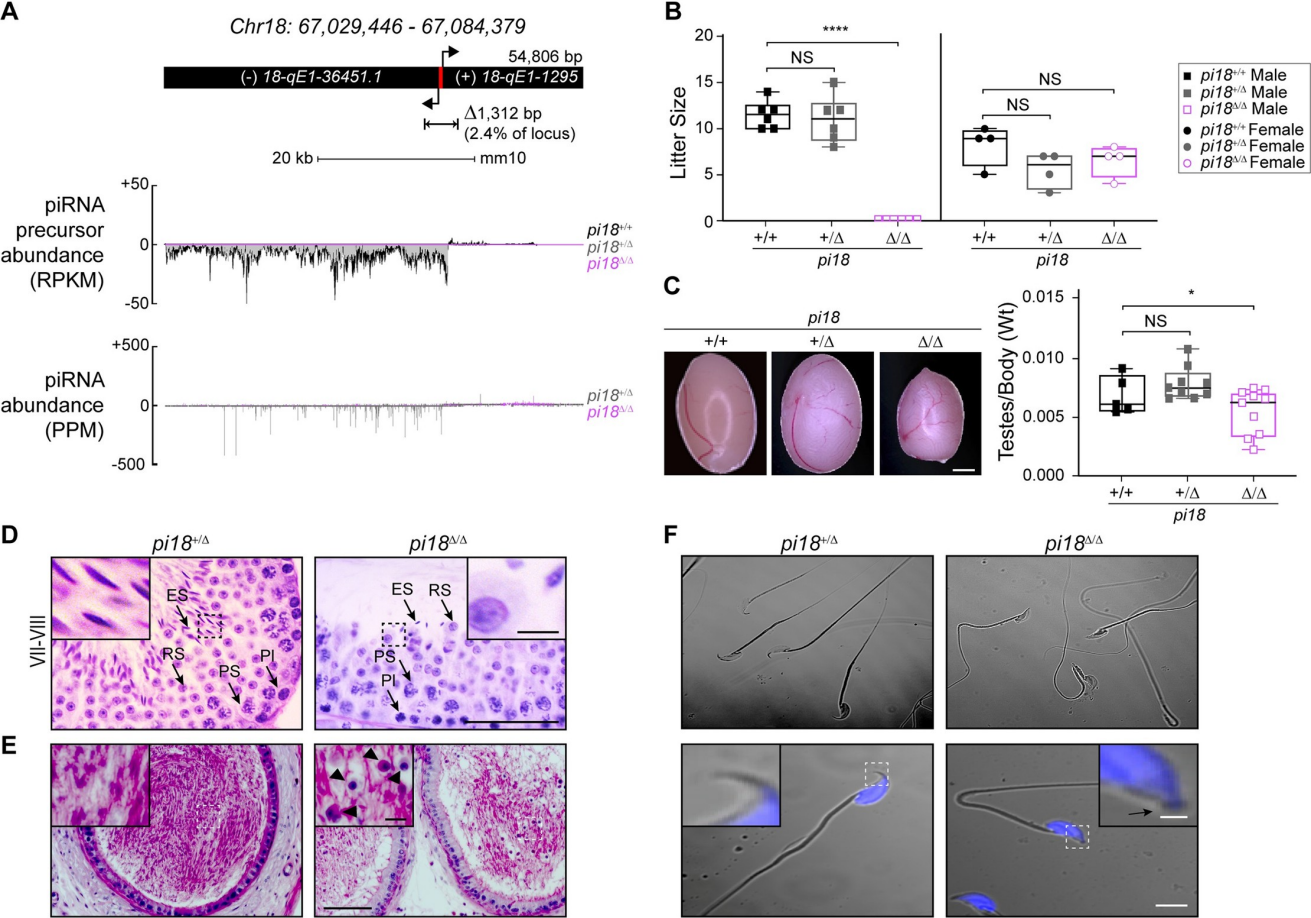
Pachytene piRNA derived from *pi18* is essential for spermatogenesis and male fertility. (A) Schematic diagram of the Chr18 piRNA coding region and the DNA fragment deleted using CRISPR/Cas9 to generate bi-directional promoter deletion (Δ/Δ) mutant mice (top). Red bar, A-MYB and BTBD18 binding loci. Precursor and mature piRNA abundance at *pi18* piRNA cluster in P28 testes of wild-type (+/+), heterozygous (+/Δ) and homozygous (Δ/Δ) mutant mice (bottom). RPKM, reads per kilobase million; PPM, parts per million reads. (B) Average litter size of adult male (squares, *n* = 6) and female (circles, *n* = 4) controls and mutant mice. (C) Representative macroscopic appearance (left) and quantification (right) of testes weight of 8 wk/old controls and mutant mice (*n* ≤ 10 per each genotype). Scale bar, 2 mm. (D) Testicular sections from 8 wk/old mice were stained with periodic acid-Schiff (PAS) and hematoxylin (H) (*n* = 3). Stage of seminiferous epithelium cycles was determined by morphology of spermatocytes and rounds spermatids. Pl, preleptotene spermatocyte; PS, pachytene spermatocyte; RS, round spermatid; ES, elongating spermatid. Scale bar, 50 μm; inset, scale bar, 5 μm. (E) PAS&H staining of cauda epididymis from 12 wk/old mice (*n* = 2). Black arrowheads, sloughing germ cells. Scale bar, 50 μm; inset, scale bar, 5 μm. (F) Representative differential interference contrast (DIC) micrograph images of sperm from 12 wk/old mice, with nuclei counterstained with Hoechst 33342 (blue) (*n* = 3). Scale bar, 5 μm; inset, scale bar, 0.5 μm; Arrow, apical hook. B, C The box indicates median ± interquartile range, the whiskers indicate the highest/lowest values and midlines are median values. NS, not significant, *P < 0.05, ****P < 0.0001.

To disrupt the bi-directional piRNA promoter at the precursor locus on Chr18, we used a pair of single-guide RNAs (S1A and S1C Fig). From founders that passed the mutant allele through their germline, we established mouse lines with a ~1.3 kb deletion in the promoter of the *pi18* piRNA cluster and bred them to homozygosity (referred to as *pi18*^*Δ/Δ*^, S1B Fig). To determine if the loss of pachytene piRNAs transcribed on Chr18 affected reproduction, we mated B6D2_F1_ females with *pi18*^*Δ/Δ*^ or control male mice. Vaginal plugs were observed in all females. *pi18*^*Δ/Δ*^ male mice did not produce litters whereas control males did and *pi18*^*Δ/Δ*^ female mice had normal fertility (Fig 1B).

The growth rates of *pi18*^*Δ/Δ*^ and control male mice did not differ, but the average weight of testes from adult mutant mice was ~45% less than controls (Fig 1C). Because *pi18*^*Δ/Δ*^ male mice were sterile, we investigated the stage at which spermatogenesis failed. Despite having half the genetic identity of WT controls, heterozygous *pi18*^*+/Δ*^ mice have WT levels of *pi18* piRNA precursors, *in vivo* fertility, and no discernable phenotype (Fig 1A, 1B, 1D and 1F). This suggests that *pi18* pachytene piRNAs are not haploinsufficient and *pi18*^*+/Δ*^ were used subsequently as controls to investigate *pi18*^*Δ/Δ*^ mutant defects. Compared to these controls, spermatocytes, round spermatids, and early elongating spermatids were largely unaltered, while condensed spermatids at steps 14–16 of spermiogenesis (stages III-VIII of spermatogenesis) were significantly reduced in the seminiferous tubules of *pi18*^*Δ/Δ*^ testes (Fig 1D). Concomitantly, there was a significant increase in the number of apoptotic cells in seminiferous tubules of *pi18*^*Δ/Δ*^ mice. Examination of seminiferous tubules indicated that a significant number of spermatocytes (but not all) become apoptotic at stage IX-X and most apoptotic cells were identified as spermatids in stage XI-XII (S2A Fig). In addition, we frequently found histological abnormalities including disordered arrangement of elongating spermatids and vacuolation in *pi18*^*Δ/Δ*^ testes (S2B and S2C Fig). *pi18*^*Δ/Δ*^ mice were infertile, but spermatozoa associated with sloughed germ cells were present in the lumen of their epididymides (Figs 1E and S2D).

### *pi18* pachytene piRNAs are essential for sperm capacitation and fertilization

**Fig 2:**
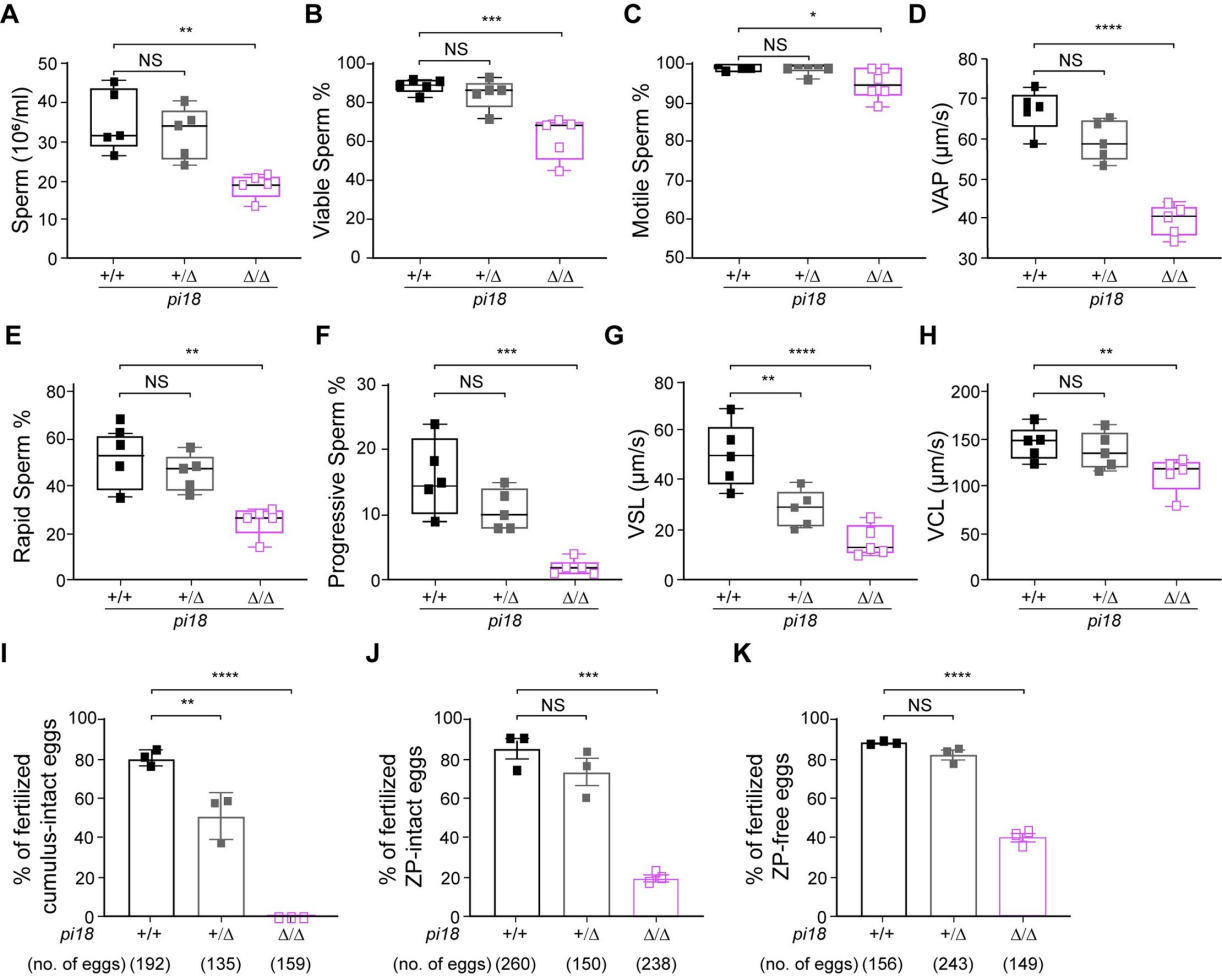
Impaired motility and *in vitro* fertilization with *pi18*^*Δ/Δ*^ sperm. (A) Sperm counts from 8 wk/old mice from *pi18*^*+/+*^ (black square), *pi18*^*+/Δ*^ (grey square), and *pi18*^*Δ/Δ*^ (purple square) mice (*n* = 5 for each genotype). (B) Sperm viability was determined by the nuclear dye Hoechst 33342 staining. The viable spermatozoa, which fluoresced pale-blue with Hoechst 33342, was expressed as a percent of the number of viable sperm/total sperm. (C) Computer-Assisted Sperm Analysis (CASA) assay of average sperm motility from 8 wk/old mice (*n* = 5 for each genotype). (D) Path velocity (velocity average path, VAP). (E) Rapid sperm motility (% of motile sperm with VAP ≥ 10 μm/s). (F) Progressive motility (% of motile sperm with VAP ≥ 50 μm/s and STR = VSL/VAP ≥ 50%, straightness, STR). (G) Linear velocity (velocity straight line, VSL). (H) Track velocity (velocity curvilinear, VCL). The box indicates median ± interquartile range, the whiskers indicate the highest/lowest values and midlines are the median values. (I) *pi18*^*Δ/Δ*^ sperm are unable to fertilize wildtype eggs *in vitro*. Controls and *pi18*^*Δ/Δ*^ sperm were inseminated with cumulus-intact eggs for 3 hours, (J) ZP-intact eggs for 3 hours and (K) ZP-free eggs for 2 hours. The average fertilization rate (mean ± s.d.) from three independent experiments is presented. Each square represents individual male mice that were used for IVF. The total number of analyzed eggs per condition is in the parenthesis. NS, not significant, *P < 0.05, **P < 0.01, ***P < 0.001, and ****P < 0.0001.

To further characterize the pathology, sperm were collected from the vas deferens and cauda epididymis of control and *pi18*^*Δ/Δ*^ mice. Intriguingly, most sperm from *pi18*^*Δ/Δ*^ mice exhibited various abnormalities of acrosomal overgrowth (Fig 1F). The number of *pi18*^*Δ/Δ*^ sperm was significantly less than controls, but 62% of mutant sperm were viable (Fig 2A and 2B). Based on computer-assisted sperm analysis (CASA), most caudal sperm from *pi18*^*Δ/Δ*^ mice were motile, but had severely decreased hyperactivity and all parameters describing the speed of their movements, including path velocity (VAP), track velocity (VCL), and linear velocity (VSL) were significantly reduced. In particular, only 2.6% of the mutant sperm had progressive motility (VAP ≥ 50 μm/s and STR = VSL/VAP ≥ 50%) (Fig 2C–2H).

*In vitro* fertilization (IVF) assays were performed to investigate the ability of *pi18*^*Δ/Δ*^ sperm to fertilize eggs. Cumulus-intact oocytes were obtained from wild-type females and inseminated with capacitated sperm from *pi18*^*Δ/Δ*^ and control mice. After 24 hours, fertilization rates were determined by the presence of two-cell embryos. Whereas 156 of 192 (81.3%) and 68 of 135 (50.4%) eggs were fertilized by sperm derived from *pi18*^*+/+*^ and *pi18*^*+/Δ*^ sperm, respectively, no fertilization was observed with *pi18*^*Δ/Δ*^ sperm (Fig 2I). Notably, although IVF was performed in media containing reduced glutathione to destabilize the extracellular zona pellucida (ZP) [32,33], eggs were not fertilized by *pi18*^*Δ/Δ*^ sperm. IVF with cumulus-intact oocytes results documented that *pi18*^*Δ/Δ*^ sperm can pass through the cumulus cell layers but fail to penetrate the zona matrix. Therefore, to define defects in *pi18*^*Δ/Δ*^ sperm more precisely, we examined sperm zona pellucida (ZP)-binding, penetration, and sperm-oocyte fusion using ZP-intact and -free oocytes, respectively. Although fertilization rates were not comparable to controls, removing the cumulus cell layers for IVF (ZP-intact: *pi18*^*+/+*^, 84.7%; *pi18*^*+/Δ*^, 73.1%; *pi18*^*Δ/Δ*^, 19.8%) versus (ZP-free: *pi18*^*+/+*^, 89.9%; *pi18*^*+/Δ*^, 81.7%; *pi18*^*Δ/Δ*^, 39.7%), significantly improved gamete fusion and fertility for *pi18*^*Δ/Δ*^ sperm (Fig 2J and 2K). We conclude that the *in vivo* and *in vitro* defects of *pi18*^*Δ/Δ*^ sperm support an essential role of *pi18* pachytene piRNAs in sperm hypermotility, zona pellucida binding and zona penetration.

### Defects of spermiogenesis and acrosome exocytosis in *pi18*^*Δ/Δ*^ mice

**Fig 3:**
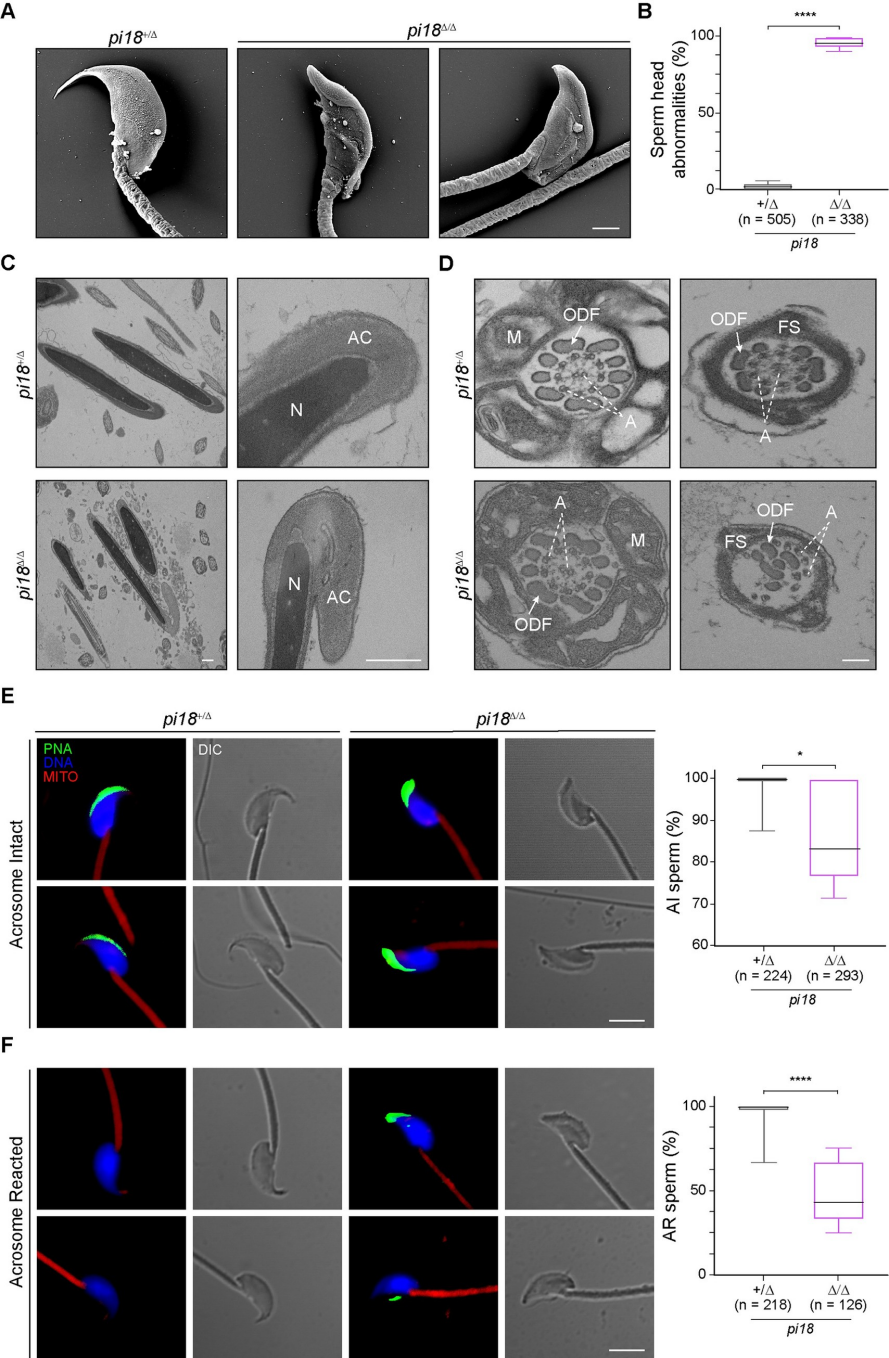
Defective spermiogenesis and impaired acrosome reaction in *pi18*^*Δ/Δ*^ mice. (A) Representative scanning electron microscopy (SEM) images of malformed sperm head in 12 wk/old control and *pi18*^*Δ/Δ*^ mutant mice (*n* = 3 per group). Scale bar, 1 μm. (B) Quantification of malformed sperm heads in A. The box indicates median ± interquartile range, the whiskers indicate the highest/lowest values and midlines are median values. (C) Transmission electron micrographs (TEM) of sperm heads from 12 wk/old mice (*n* = 4 for each genotype). Scale bar, 0.5 μm; AC, acrosome; N, nucleus. (D) TEM images of sperm mid (left) and principal (right) pieces. Scale bar, 0.5 μm; ODF, outer dense fiber; M, mitochondria; A, 9+2 axoneme; FS, fibrous sheath. (E) Representative confocal images (left) and quantification (right) of acrosome-intact cauda epididymal sperm from 12 wk/old control and mutant mice. Sperm were stained for fluorescent-dye-labeled peanut agglutinin (PNA) (acrosome, green); MitoTracker Red FM (mitochondria, red); Hoechst 33342 (DNA, blue). Scale bar, 5 μm; AI, acrosome intact; AR, acrosome reacted; DIC, differential interference contrast (*n* = number of sperm total from 3 independent experiments). The box indicates median ± interquartile range, the whiskers indicate the highest/lowest values and midlines are median values. (F) Same as (E) by for acrosome-reacted sperm. Acrosome exocytosis was induced with calcium ionophore A23187. *P < 0.05, ****P < 0.0001.

Scanning (SEM) and transmission electron microscopy (TEM) of sperm heads, acrosomes and cross-sections along the length were used to more precisely characterize sperm dysmorphology. The majority of *pi18*^*Δ/Δ*^ sperm heads (94.8% of mutant sperm) were abnormally round shaped with shortened apical hooks, smaller apical angles and bulges in the acrosome region (Fig 3A and 3B). Although all layers, including plasma, outer and inner acrosomal as well as nuclear membranes were intact, the dramatic overgrowth of the acrosome excessively folded onto itself (Fig 3C). Cross sections of the mid-piece of *pi18*^*Δ/Δ*^ sperm document a well-defined mitochondrial sheath, normally arranged outer dense fibers (ODF) and an axoneme with an intact “9+2” microtubule structure. However, the axonemal complex was abnormal, and the outer dense fibers were unassembled in the principal piece (tail) of *pi18*^*Δ/Δ*^ sperm (Fig 3D). The outer dense fibers (ODFs) are prominent sperm tail-specific cytoskeletal structures and are thought to be contractile in the sperm tail [34,35]. Among them, ODF2 is a major component of ODFs. *Odf2*^*Null*^ spermatozoa display marginal defects in mid- and principal pieces and the absence of ODF2 results in abnormal motility and bent tails [36]. Our proteomic analysis identified altered ODF2 protein levels, which could account for the severely reduced hyperactivity and deformed structural defects in *pi18*^*Δ/Δ*^ sperm (S6 and S7 Tables).

To further investigate the inability of *pi18*^*Δ/Δ*^ sperm to fertilize eggs, we used Alexa Fluor 488-conjugated peanut agglutin (PNA) to determine acrosome exocytosis which is a prerequisite for gamete fusion [24]. PNA binds to the outer acrosomal membrane and, in agreement with previous results [37–39], fluorescent staining on the crescent region of acrosome-intact *pi18*^*+/Δ*^ control sperm disappeared after induction of the acrosome exocytosis by calcium ionophore, A23187. The dorsal edge of the acrosome is not fully elongated in *pi18*^*Δ/Δ*^ sperm and, consistent with SEM and TEM images, exhibited accumulated PNA fluorescence on the acrosomal vesicle bulge. Notably, about half of *pi18*^*Δ/Δ*^ sperm had slightly reduced PNA signal on their acrosomes which did not disappear after induction of acrosome exocytosis (Fig 3E and 3F). Taken together, differences in sperm velocities and impaired acrosomal reaction contribute to the inability of *pi18*^*Δ/Δ*^ sperm to fertilize wildtype eggs.

### Altered acrosome formation in *pi18*^*Δ/Δ*^ spermatids

To link development of acrosome abnormalities to the phase of spermiogenesis, we used light microscopy to examine spermatogenic cells from control and *pi18*^*Δ/Δ*^ mice. The Golgi apparatus of spermatids consists of several stacks of saccules with a *cis*-network facing the endoplasmic reticulum (ER), and a *trans*-Golgi network (TGN) facing the nuclear envelope. Budding proacrosomal vesicles (PVs) from the TGN initiate acrosome formation and proper trafficking from the TGN toward the nucleus is essential for normal shaping and sizing of the acrosome. Periodic acid-Schiff (PAS) and PNA staining documented the presence of normal-shaped acrosomes in the Golgi phase of *pi18*^*Δ/Δ*^ spermatids and there were no obvious differences with control spermatids (S3A and S3B Fig, upper panels).

**Fig 4:**
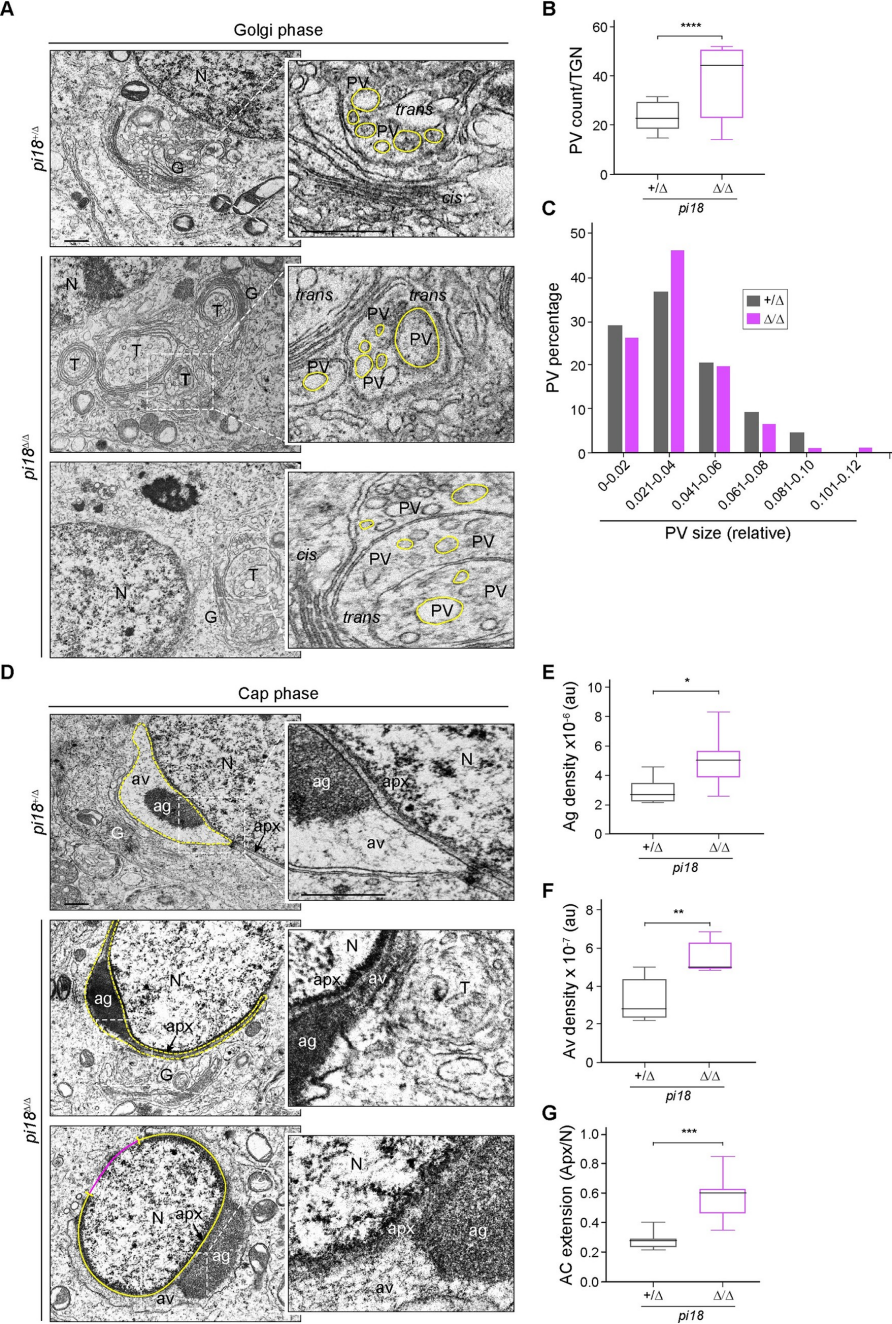
Deformed acrosome formation from disrupted proacrosomal vesicles in *pi18*^*Δ/Δ*^ mice. (A) Representative TEM images of proacrosomal vesicles in Golgi phase round spermatids in 12 wk/old control and mutant mice (*n* = 2 per group). Enlarged insets (right) correspond to the dashed box (left) of the Golgi apparatus. Scale bar, 0.5 μm; G, Golgi apparatus; T, *trans*, and TGN, *trans*-Golgi network; *cis*, *cis*-Golgi network; PV, proacrosomal vesicle; N, nucleus. (B) Quantification of proacrosomal vesicles (PV) in A. The box indicates median ± interquartile range, the whiskers indicate the highest/lowest values and midlines are median values. (C) PV size distribution determined by measuring diameters in control and mutant round spermatids at the Golgi phase in A. The diameters are presented in relative units. PV sizes are binned as indicated. (D) TEM images of cap phase round spermatids in 12 wk/old control and mutant mice (*n* = 2 for each genotype). The acrosome at cap phase round spermatids in dashed boxes (left) are enlarged (right). Scale bar, 0.5 μm; Ac, acrosome; Ag, acrosomal granule; Av, acrosomal vesicle; Apx, acroplaxome. (E) Quantification of acrosomal granule density AU, arbitrary unit. The box indicates median ± interquartile range, the whiskers indicate the highest/lowest values and midlines are median values. (F) Same as (E), but for acrosomal vesicle density. (G) Same as (E), but for acrosome extension. *P < 0.05, **P < 0.01, ***P < 0.001, and ****P < 0.0001.

However, using TEM in control mice, we observed umbrella shaped TGNs and multiple PVs of uniform size located between the TGN and the nuclear membrane in control spermatids. Although PVs were present in *pi18*^*Δ/Δ*^ spermatids, they were not uniform in size and appeared larger than those in control sperm. Moreover, we frequently observed that the lamellar structure of the TGN formed loose whorls in *pi18*^*Δ/Δ*^ spermatids (Fig 4A). These observations suggested abnormalities in the vesicles budding from the TGN in *pi18*^*Δ/Δ*^ spermatids. To quantify the foregoing observations, we counted and measured the diameter of PVs on the TEM sections (Figs 4A and S4A). *pi18*^*Δ/Δ*^ spermatids tend to produce more PVs with larger diameters than vesicles in control spermatids (Fig 4B and 4C). These results indicate that aberrant PV formation and budding from the TGN resulted in formation of deformed acrosomes in *pi18*^*Δ/Δ*^ spermatids.

As spermiogenesis proceeded to the cap phase, the defects in *pi18*^*Δ/Δ*^ spermatids became more obvious. In the cap phase, proacrosomal vesicles (PV) fuse with each other to form the acrosome granule (AG) at the acroplaxome (Apx) which anchors the acrosome (Ac) to the nuclear membrane over which the acrosome flattens [40]. In control spermatids stained with PNA, the acrosome grew into a single cap-like structure that covered nuclei (S3A and S3B Fig, middle panels). In contrast, *pi18*^*Δ/Δ*^ spermatids displayed a notably enlarged acrosomal cap composed of highly electron dense acrosomal granules and vesicles. Aberrant PV formation in the Golgi phase inundated the acrosome with vesicles, and as determined by the marginal ring of the Apx, the nucleus became covered with an overgrown acrosome at the cap phase in *pi18*^*Δ/Δ*^ spermatids (Figs 4D–4G and S4B). In the subsequent acrosome phase of spermiogenesis, plump and thickening acrosomes were observed in *pi18*^*Δ/Δ*^ spermatids, but the positioning of the perinuclear ring of the manchette, a transient microtubular/actin-containing structure [27], was comparable to controls (S3A and S3B, lower panels, and S4C Figs). Together, these results indicate that *pi18* pachytene piRNAs play an essential role in regulating acrosome biogenesis during spermiogenesis.

### *pi18* pachytene piRNAs control *Golga2* mRNA abundance

**Fig 5:**
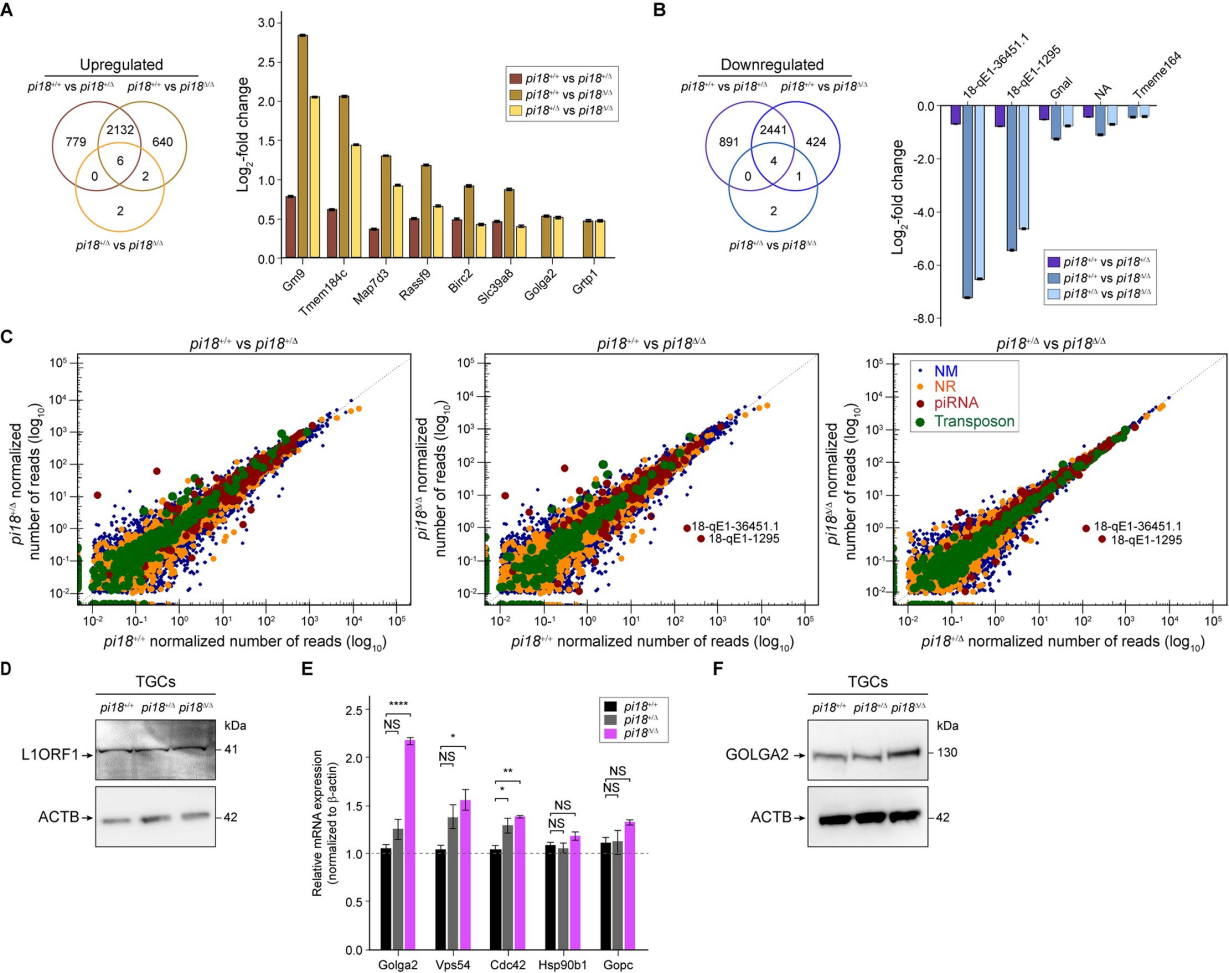
Altered abundance of *Golga2* in *pi18*^*Δ/Δ*^ mice. (A) Venn diagrams depicting the overlap of up-regulated (left) in wild-type (+/+) vs heterozygous (+/Δ), wild-type (+/+) vs homozygous (Δ/Δ), and heterozygous (+/Δ) vs homozygous (Δ/Δ) of RNA-seq data from *pi18*^*+/+*^, *pi18*^*+/Δ*^, and *pi18*^*Δ/Δ*^ testes (*n* = 3 for each genotype) at P28 using adjusted *P* < 0.01 as the cut off. Up-regulated (log_2_-fold change) in Venn diagrams (right). (B) Same as (A), but for down-regulated genes (left) and down-regulated (log_2_-fold change) in Venn diagrams (right). (C) Scatter plots comparing +/+ to +/Δ (left), +/+ to Δ/Δ (middle) and +/Δ to Δ/Δ (right) of RNA-seq reads assigned to mRNA (NM, blue); non-coding RNA (NR, orange); piRNA (red); transposons (green). (D) Immunoblot of L1ORF1 in testicular germ cells (TGCs) from *pi18*^*+/+*^, *pi18*^*+/Δ*^, and *pi18*^*Δ/Δ*^ (*n* = 3 for each genotype). Actin is used as a load control. Image is representative of three independent experiments. (E) Quantitative RT-PCR validation of up-regulated genes related to acrosome biogenesis using *β-actin* transcript as an internal control (*n* = 3 per genotype). Data are shown as mean ± s.d. Results shown reflect three independent experiments. (F) Same as (D), but for GOLGA2. NS, not significant, *P < 0.05, **P < 0.01, and ****P < 0.0001.

To investigate molecular consequences in *pi18*^*Δ/Δ*^ mice, we isolated mRNA and small RNA from controls and *pi18*^*Δ/Δ*^ testes at P28 and performed RNA-seq. The first wave of spermatogenesis is complete by P35 [41] and pachytene piRNA expression peaks at P17.5 [5]. Therefore, to avoid testicular sperm contamination and examine the end-point transcriptional alterations in spermatocytes and spermatids, we used testes from controls and *pi18*^*Δ/Δ*^ at P28. The mRNA abundance in *pi18*^*Δ/Δ*^ testes was extensively altered compared to *pi18*^*+/+*^ controls. In contrast, *pi18*^*Δ/Δ*^ mutants had modest but significant changes in mRNA abundance compared with *pi18*^*+/Δ*^ controls (Figs 5A, 5B and S5A). RNA-seq analyses identified 6 up-regulated and 4 down-regulated genes in *pi18*^*Δ/Δ*^ testes compared with *pi18*^*+/+*^ and *pi18*^*+/Δ*^ controls (Fig 5A and 5B and S1–S3 Tables; *P* < 0.01, FDR < 0.1). However, the steady-state abundance of piRNA precursors was unaffected outside the *pi18* piRNA cluster (Fig 5B). We also investigated whether pachytene piRNA ablation forced transposon de-repression in *pi18*^*Δ/Δ*^ testes. The ordinary abundance of transposon RNA in *pi18*^*Δ/Δ*^ testes documented that the fertilization and spermiogenic defects do not reflect a failure to silence transposons (Fig 5C and S4 Table). In addition, we found steady-state express of the major class of retrotransposons, LINE 1 element protein level in *pi18*^*Δ/Δ*^ testes (Fig 5D). Together with small RNA-seq (S5B Fig), these analyses document that deletion of the bi-directional promoter at the *pi18* pachytene piRNA cluster eliminates precursor and processed piRNAs encoded at the site without an accompanying effect on other piRNA clusters or transposons.

Despite the severely deformed sperm head morphology, the most enriched transcripts in gene ontology (GO) analysis of *pi18*^*Δ/Δ*^ mice related to chromosome segregation, DNA repair, and meiotic recombination (S5C Fig and S5 Table). However, immunostaining of meiotic chromosome spreads of *pi18*^*Δ/Δ*^ were indistinguishable from *pi18*^*+/Δ*^ spermatocytes, the DNA damage marker γH2AX expression was confined to the sex body, and successful synapsis and recombination was observed in the absence of *pi18* piRNAs (S5D Fig). Therefore, we further searched annotated gene function that might be related to the observed head dysmorphology in *pi18*^*Δ/Δ*^ mice. Among transcripts that were up-regulated in mutant mice, we identified *Golga2* that encodes a *cis*-side localized Golgi matrix protein (Fig 5A). It has previously been reported that the absence of GOLGA2 in gene-edited mice results in male infertility [42]. These mutant mice, lack acrosomes, have round sperm heads and mitochondrial defects similar to human globozoospermia which is opposite to the acrosomal overgrowth phenotype observed in *pi18*^*Δ/Δ*^ mice. In addition, over-expression of GOLGA2 in heterologous cells results in irregular and incorrectly aligned stacks of abnormally elongated Golgi including bending and horseshoe-like structures which are similar with *pi18*^*Δ/Δ*^ spermatids [43].

In analyzing TMT mass spectrometry data, we found a correlation between mRNA and protein expression level of GOLGA2 in *pi18*^*Δ/Δ*^ testes and validated this finding by immunoblot (Figs 5F and S6A and S6 and S7 Tables). Compared to *pi18*^*+/+*^ and *pi18*^*+/Δ*^ controls, the level of GOLGA2 protein was higher in *pi18*^*Δ/Δ*^ testicular germ cells (TGCs) and this increase does not reflect a failure of cytoplasmic depletion in *pi18*^*Δ/Δ*^ spermatids during spermiogenesis. We observed GOLGA2 movement from the Golgi near the overgrown acrosome at the cap phase to the depleted cytoplasm at the acrosomal phase of *pi18*^*Δ/Δ*^ spermatids (S6B Fig). Previous investigations reported that the absence of GOLGA2 did not affect PV secretion and thus, the piRNAs absent in *pi18*^*Δ/Δ*^ spermatids must reflect additional molecular defects to account for the observed aberrant PV and TGN during acrosome biogenesis. Transcriptome analysis documented that loss of *pi18* piRNA does not have a significant effect on the abundance of piRNA pathway transcripts and only affects post-transcriptional silencing during the final steps of differentiation in mouse spermiogenesis. Of note, other selected transcripts involved in acrosomal vesicle trafficking and fusion also were significantly increased in *pi18*^*Δ/Δ*^ testes compared with *pi18*^*+/+*^ and *pi18*^*+/Δ*^ controls (Fig 5E). However, they did not have 3’UTR piRNA seed sequences corresponding to the annotated *pi18* piRNAs, suggesting that other mechanisms may pertain. As noted, RNA abundance in *pi18*^*+/Δ*^ controls are comparable to WT controls. Therefore, the *pi18*^*+/+*^ vs. *pi18*^*+/Δ*^ and *pi18*^*+/+*^ vs. *pi18*^*Δ/Δ*^ comparisons representing the massive mRNAs (2132 of up-and 2441of down-regulated) appear to have no *pi18* targeting and only Golga2 appears to be related to the *pi18*^*Δ/Δ*^ acrosomal defects. A previous study concluded that the vast majority of *pi6* piRNAs appear to have no regulatory targets. Indeed, only six mRNAs appear to be direct *pi6* piRNA targets [23].

## Discussion

We have previously reported that BTBD18, a nuclear protein expressed in male germ cells, acts as a licensing factor required for transcriptional elongation of precursor transcripts at 50 of the 115 pachytene piRNA clusters present in the mouse genome. *Btbd18*^*Null*^ spermatids undergo meiosis and arrest in the Golgi phase of spermiogenesis (stages 1–3). Elongated spermatids are not observed, and male germ cells undergo apoptosis [31]. We anticipated that ablation of individual binding sites of BTBD18 in the mouse genome would phenocopy a subset of *Btbd18*^*Null*^ abnormalities. Indeed, disruption of the single pachytene piRNA producing locus on Chr18 resulted in notable phenotypic defects and male sterility. Thus, the severe defects observed in *pi18*^*Δ/Δ*^ mice appear to reflect a low degree of functional redundancy with other piRNA clusters.

The relatively late expression of pachytene piRNA suggests a major role in regulating post-meiotic spermiogenesis [18,19]. Despite *Miwi*^*Null*^ mice failure to generate pachytene piRNA, round spermatids are generated, which indicates that meiosis can be completed without MIWI and MIWI-binding pachytene piRNAs [44,45]. Instead, the main defect of *Miwi*^*Null*^ mice is failure of haploid sperm differentiation that results in the absence of mature spermatozoa. Unlike *Miwi*^*Null*^ and *Btbd18*^*Null*^ mice, *pi18*^*Δ/Δ*^ mice can produce mature spermatozoa but are infertile. Consistent with predictions, the molecular defects of *pi18*^*Δ/Δ*^ mice occur during spermiogenesis in the transformation of haploid round spermatids to mature, elongated spermatozoa. Spermatogenesis was normal in *pi18*^*Δ/Δ*^ mice until the onset of acrosome biogenesis at the Golgi phase of spermiogenesis when sperm dysmorphology was associated with abnormalities in transcript and protein abundance.

The *pi18* piRNA cluster is one of the highest piRNA-producing loci among all (>200) pre-and pachytene piRNA clusters [23,46]. While *pi18*^*+/Δ*^ control mice had normal fertility and no discernable phenotype, transcriptome analysis revealed minor changes of mRNA abundance between *pi18*^*+/Δ*^ and *pi18*^*Δ/Δ*^ testes which suggests compensatory mechanisms that could partially account for the difficulty in identifying pachytene piRNA targets and function [22,47]. Ablation of the promoter of the single bi-directional site on *pi18* causes a phenotype that develops later in spermiogenesis compared to *Btbd18*^*Null*^ mice. *pi18*^*Δ/Δ*^ spermatids can elongate albeit with severe head dysmorphology that affects acrosome exocytosis and poor progressive motility that render male mice infertile. Comparison of the transcriptomes of *pi18*^*Δ/Δ*^ testes with *pi18*^*+/+*^ and *pi18*^*+/Δ*^ controls suggests that removal of a subset of piRNAs can increase mRNA abundance reflecting disruption of RNA homeostasis. However, how *pi18* piRNAs recognize and regulate their targets to ensure successful spermiogenesis will need further exploration. Previous studies have documented that exogenous human piRNA from a transgene in mouse and endogenous mouse piRNA from chromosome 6 (*pi6*) direct the cleavage and repression of a specific target mRNA [19,23]. This raises the possibility that *pi18* pachytene piRNAs may have mRNA and piRNA precursors as direct cleavage targets which could be explored in the future by degradome sequencing [48].

The primary alteration in spermiogenesis in *pi18*^*Δ/Δ*^ mice was loss of structural integrity of the trans-Golgi network (TGN) which appeared as loose whorls associated with enlarged proacrosomal vesicles (PV). The loss of *pi18* pachytene piRNAs induced enhanced PV formation and trafficking that resulted in dramatic acrosomal overgrowth. Although post-transcriptional repression of mRNA targets by miRNAs is well documented [49], a potential role for pachytene piRNAs in targeting specific RNAs for degradation also has been reported [18,19,23]. A previous study [50] reported that piRNAs could act through seed complementarity using 7mer seed matches like miRNA. According to this mechanism, each abundant piRNA could have >100 regulatory targets. Instead, similar to previous findings [23], the loss of *pi18* pachytene piRNAs only affects a small number of mRNA and proteins. This suggests that pachytene piRNAs may represent a novel class of selfish genetic elements whose maintenance is assured by positive selection for a small number of pachytene piRNA-directed regulatory events [23,51].

Several gene-edited mouse models, including *Golga2*^*Null*^ mice, with defects in acrosome biogenesis result in loss of acrosome formation and globozoospermia which is a phenotype seen in infertile humans [39,42,52–59]. During the Golgi phase of spermiogenesis in *Smap2*^*Null*^ mice (lacking an arf GTPase-activating protein), spermatids had similar defects of PV formation and distorted TGN structure and yet produced globozoospermia [55]. In addition, in the absence of zona pellucida binding protein 2, *Zpbp2*^*Null*^ spermatozoa have subtle head deformations with shortened apical hooks and bulges but were still able to undergo acrosome exocytosis [54]. Moreover, in the absence of proprotein convertase 4, *Pcsk4*^*Null*^ mice had a sickle-shaped head and lacked the pointed apex similar with *pi18*^*Δ/Δ*^ sperm. However, mRNA abundance and protein level of PCSK and its substrate, acrosin-binding protein, ACRBP were not altered in *pi18*^*Δ/Δ*^ mice [56]. Therefore, the observed acrosomal overgrowth and impaired acrosome exocytosis appear unique to *pi18*^*Δ/Δ*^ mice and reflect the functional significance of *pi18* pachytene piRNAs as a regulator for acrosome biogenesis during the spermiogenesis. The *pi18*^*Δ/Δ*^ mice will be useful to investigate molecular mechanisms by which pachytene piRNAs regulate mRNA abundance to ensure production of functional spermatozoa and successful fertilization.

## Materials and methods

### Ethics statement

All experiments with mice were conducted in accordance with guidelines of the National Institute of Health under a Division of Intramural Research and NIDDK Animal Care and Use Committee approved animal study protocol (protocol numbers KO18-LCDB-18 and KO44-LCDB-19).

### Generation of CRISPR/Cas9 mutant mice

To establish *pi18*^*Δ/Δ*^ mutant mice, single guide RNA (sgRNA) sequences were designed to target the bi-directional promoter and flanking sequence of the *pi18* piRNA cluster. Synthetic double-stranded DNA was cloned into pDR274 (Addgene, #42250) to express sgRNA. After digestion with *Dra*I, the linearized DNA fragment was purified with a PCR Clean-up Kit (Clontech Laboratories) and *in vitro* transcribed using the AmpliScribe T7-Flash Transcription Kit (Lucigen). *Cas9* cRNA (Addgene #42251) was generated after linearization with *Pme*I, purified with the PCR clean-up kit, and *in vitro* transcribed with mMESSAGE mMACHINE T7 (Thermo Fisher Scientific). Both sgRNA and *Cas9* cRNA were purified with MEGAclear Transcription Clean-Up Kit (Thermo Fisher Scientific). To collect zygotes from oviducts at embryonic day 0.5 (E0.5), hormonally stimulated B6D2_F1_ (C57LB/6 × DBA2) female mice were mated with B6D2_F1_ male mice. Mixed sgRNA (50 ng/μl) and *Cas9* cRNA (100 ng/μl) were injected into zygotes in M2 medium. Injected zygotes were cultured (12–18 hr) in KSOM (37°C, 5% CO_2_) supplemented with 3 mg/ml BSA to two-cell embryos and transferred into oviducts of pseudo-pregnant ICR mice. To determine the genotype of mutant founders, genomic DNA was extracted from tail tips and lysed in 150 μl of DirectPCR Lysis Reagent (Viagen Biotech) with protease K (0.2 mg/ml, Thermo Fisher Scientific) at 55°C for 5 hr. Following protease K inactivation by incubation at 85°C for 1 hr, samples were genotyped by PCR. After purification, PCR products were cloned into TOPO blunt vectors for DNA sequencing. Mouse mutant lines were established and maintained by mating mutant founders with B6D2_F1_ females or males. All mutant mice in this study were backcrossed for at least two generations before use.

### Mouse sperm preparation

Sperm from cauda epididymides were released into Cook medium (Cook Medical) and squeezed from vas deferens.

### Fertility

To assess fertility, individual 2–8 mo old male mice were co-caged with two B6D2_F1_ females for 2 wk to 6 mo. The average number of pups per litter was quantified and at least 5 mating cages were set up for each genotype. Female mice were checked for the presence of vaginal plugs and pregnancy. The same procedures were used to assess the fertility of *pi18*^*Δ/Δ*^ and control female mice.

### *In vitro* fertilization

To assess *in vitro* fertility, caudal epididymal sperm were isolated from 2–8 mo old *pi18*^*Δ/Δ*^ and control mice and capacitated for 1.5 to 2 hr in 0.5 ml of Cook medium (Cook Medical). Wild-type B6D2_F1_ female mice (2–3 mo old) were synchronized with 5 U of PMSG and induced to ovulate with 5 U of hCG administered 48 hr later. Cumulus-intact eggs were recovered from oviducts 15 to 16 hr later in 0.2 ml of Cook medium with 1 mM reduced glutathione (GSH, Sigma). To minimize differences in the quality of recovered eggs, cumulus-intact eggs in one oviduct were separated from those in the other oviduct. To investigate zona pellucida (ZP) binding and penetration, cumulus cells were removed by incubating in 0.3 mg/mL hyaluronidase (Sigma). Capacitated sperm (1.5 X 10^5^/ml) were added to each pool and incubated for 3–6 hr (37°C, 5% CO_2_ in air). The eggs were transferred to KSOM (37°C, 5% CO_2_) supplemented with 4 mg/ml BSA and the presence of two pronuclei was recorded as fertilized. For the sperm-oocyte membrane fusion assays, the ZP was dissolved by treating the eggs with acid Tyrode solution (Sigma) for 10–20 seconds. ZP-free eggs were inseminated with capacitated sperm and co-incubated for 2 hours. After PBS washing, eggs were stained with Hoechst 33342, mounted on slides, and finally analyzed under an LSM 780 confocal/multiphoton microscope (Carl Zeiss). Eggs were considered fertilized when at least one decondensed sperm nucleus or two pronuclei were observed in the egg cytoplasm.

### Sperm count, motility, and morphology

To count sperm, cauda epididymides and vas deferens were harvested in pre-warmed (37°C) Cook medium (Cook Medical). 20 μl of a sperm suspension was diluted in 500 μl of Cook medium and counted in a hemocytometer using an AxioPlan 2 (Carl Zeiss) microscope. Isolated sperm motility was determined by computer assisted sperm analysis (CASA) of path velocity (VAP), straight velocity (VSL), curvilinear velocity (VCL) using HTM-IVOS (Version 12.3) motility analyzer (Hamilton Thorne). Sperm were further observed for morphological changes by light microscopy after staining for DNA with Hoechst 33342. In addition, acrosome exocytosis of the isolated sperm was induced by 20 μM calcium ionophore (A23187, Sigma Aldrich) in pre-warmed HTF media followed by incubation (37°C, 5% CO_2_, 90 min).

### Isolation of testicular germ cells (TGCs)

Testes were excised to remove seminiferous tubules which were minced gently with fine scissors (3–4 min) in 1 ml of DMEM F12 medium (Invitrogen). The minced tissue was treated with 0.05% collagenase/trypsin [60]. The cell suspensions were washed with and resuspended in DMEM F12 medium. Cell suspensions were subjected to immunoblot analysis or spread on poly-lysine-coated glass slides (15 min, RT) and then fixed in cold methanol (-20°C, 15 min). The slides were dried for 10 min to evaporate methanol, treated with 0.2% Triton X-100 for 10 min followed by four washings with PBS. The slides were placed in blocking buffer (PBS containing 3% goat serum, 1% glycerol, 0.1% BSA) for 60 min at RT and incubated overnight with primary antibodies at RT. After washing with PBS twice for 10 min, samples were incubated with secondary Alexa Fluor conjugated antibody for 60 min. The slides were mounted using antifade mounting medium and images were obtained with an LSM 780 confocal/multiphoton microscope (Carl Zeiss).

### Histology, TUNEL, immunofluorescence and confocal microscopy

Mouse testes and epididymides were incubated in Bouin’s fixative overnight at room temperature (RT) and washed with 70% EtOH. Paraffin embedded samples were sectioned (5 μm) and mounted on slides prior to staining with periodic acid-Schiff (PAS) and hematoxylin. Stages of spermatogenesis and steps of spermatid development were determined [61]. Terminal deoxynucleotidyl transferase-mediated deoxyuridine triphosphate (TUNEL) assays were used to determine apoptosis with an *In Situ* Apoptosis Detection Kit (Millipore) according to the manufacturer’s instructions. Bright field images were obtained with an AxioPlan 2 microscope (Carl Zeiss).

After deparaffinization, rehydration, and antigen retrieval with 0.01% sodium citrate buffer (pH 6.0) (Sigma Aldrich), tissue sections were incubated with blocking buffer (3% goat serum, 0.05% Tween-20, RT for 1 hr) followed by addition of primary antibodies (S9 Table) overnight at RT. Specific Alexa Fluor secondary antibodies were used to detect primary antibodies and DNA was stained with Hoechst 33342. Fluorescent images were captured with an LSM 780 confocal/multiphoton microscope.

### Meiotic chromosome spreads

To obtain meiotic chromosome spreads, de-capsuled mouse testes were incubated in hypotonic extraction buffer (30–60 min, on ice) and seminiferous tubules were chopped to release germ cells [62]. A drop of sucrose solution containing germ cells was placed on a glass slide coated with 1× PBS containing 1% PFA and 0.15% (v/v) Triton-X100 (pH 9.2) and spread by swirling. Slides were placed in a humidifying chamber (2.5 hr), air-dried, washed twice with 1× PBS with 0.4% Photo-Flo 200 solution (Electron Microscopy Science) and air-dried. For immunostaining of meiotic chromosomes, slides were blocked with blocking buffer (3% goat serum, 0.05% Tween-20, RT for 1 hr). The slides were then incubated with primary antibodies in a humidifying chamber overnight at RT. The slides were incubated with Alexa Fluor secondary antibodies (1 hr, RT). Images were obtained with an LSM 780 confocal/multiphoton microscope.

### Scanning and transmission electron microscopy

For scanning electron microscopy (SEM), sperm from cauda epididymides and vas deferens were isolated in 0.1 M phosphate buffer, pH 7.4. Sperm were attached to a poly-L-lysine coated glass coverslip and fixed (2.5% glutaraldehyde, 1% formaldehyde, 0.12 M sodium cacodylate buffer, pH 7.4, 1 hr, RT). Samples were washed in cacodylate buffer, post-fixed (1% osmium tetroxide, 1 hr) dehydrated in a graded ethanol series, and dried out of CO_2_ in a Samdri-795 critical point dryer (Tousimis Research Corp). Samples were mounted on SEM stubs with carbon adhesive, sputter-coated with 5–10 nm of gold in an EMS 575-X sputter coater (Electron Microscopy Sciences) and examined with a ZEISS Crossbeam 540 SEM at the NHLBI Electron Microscopy Core.

For transmission electron microscopy (TEM), testes, cauda epididymides, and vas deferens sperm were fixed (2.5% glutaraldehyde, 1% formaldehyde, 0.12 M sodium cacodylate buffer, pH 7.3), cut into 1 mm^3^ pieces, post-fixed (1% osmium tetroxide) and stained (1% uranyl acetate). Samples were dehydrated and embedded in Epon 812 resin. Ultrathin sections were counterstained with uranyl acetate and lead citrate. Images were acquired with a JEM 1200EX TEM equipped with an AMT 6-megapixel digital camera at the NHLBI Electron Microscopy Core. Densitometric quantification of acrosomal granules and acrosomal vesicles and diameter of nuclei were processed with ImageJ software (NIH).

### Protein extraction and immunoblots

Testicular germ cell proteins were extracted in 1× LDS sample buffer with 1× NuPAGE Sample Reducing Agent (Thermo Fisher Scientific). Proteins were separated on 4–12% Bis-Tris gels and electrophoretically transferred to PVDF membranes. The membranes were blocked with 5% nonfat milk in Tris-buffered saline containing 0.05% Tween-20 (TBS-T) at RT for 1 hr and probed with primary antibodies (S9 Table) overnight at 4°C. The membranes were washed three times with TBS-T and incubated for 1 hr at RT with secondary antibodies followed by washing with TBS-T and developed using SuperSignal West Dura Extended Duration Substrate (Thermo Fisher Scientific). Signals were detected with PXi Touch (Syngene) according to the manufacturer’s instructions. For immunoblot analysis, sperm from the cauda epididymides and vas deferens were directly released into PBS. The collected sperm were washed with PBS and then resuspended in sample buffer containing 3% SDS and boiled for 10 min.

### RNA-seq library preparation

Total RNA (100–1000 ng) was isolated from testes using miRNAeasy Mini (Qiagen) and RNA-seq libraries were constructed using TruSeq Stranded Total RNA Kit (Illumina) with Ribo-Zero following the manufacturers’ instruction. The fragment size of RNA-seq libraries was verified using a 2100 Bioanalyzer (Agilent) and concentrations were determined using Qubit (LifeTech). The libraries were loaded onto the Illumina HiSeq 3000 for 2x50 bp paired end read sequencing at the NHLBI DNA Sequencing and Genomics Core Facility. The fastq files were generated using the bcl2fastq software for further analysis.

### Small RNA-seq library preparation

As previously described for preparation of small RNA-seq libraries [63], total testes RNA (20 μg) was incubated with 4 μl of 5X borate buffer (148 mM borax, 148 mM boric acid, pH 8.6, Thermo Fisher Scientific) for 10 min at RT with 2.5 μl of freshly dissolved 200 mM NaIO_4_ (Thermo Fisher Scientific) for β-elimination. To quench unreacted NaIO_4_, 2 μl of glycerol (ThermoFisher Scientific) was added and incubated for 10 min at RT. After adding 380 μl of 1X borate buffer, RNA was precipitated with ethanol for 1 hr, at -80°C. Following centrifugation, the RNA was dissolved in 50 μl of 1X borax buffer (30 mM borax and 30mM boric acid, 17.5 mM NaOH, pH 9.5) and incubated for 90 min at 45°C prior to addition of 450 μl of 1X borate buffer and 20 μg of glycogen. The RNA was precipitated with ethanol for 1 hr, at -80°C, collected by centrifugation and dissolved in water. During β-elimination, periodate-reacted RNAs were shortened by 1 nt at the 3’ end with monophosphates and were unable to be amplified during library preparation. Thus, piRNAs, protected from β-elimination by 2’-O-methylation at the 3’ end, were enriched in the small RNA-seq libraries. For small RNA-seq library construction, NEBNext Multiplex Small RNA Library Prep Set for Illumina (New England BioLabs) was used per the manufacturer’s instructions. In general, 1 μg total RNA was subjected to 3’ and 5’ adapter ligation, reverse transcribed, PCR amplified, followed by size selection with AMPure XP beads (Beckman Coulter) for deep sequencing at the NIDDK Genomics Core Facility.

### RNA-seq data analysis

Raw sequence reads were trimmed with cutadapt 1.18 to remove any adapters while performing light quality trimming with parameters "-a ATCGGAAGAGC -A ATCGGAAGAGC -q 20—minimum-length = 25." Sequencing library quality was assessed with fastqc v0.11.8 with default parameters [64] and trimmed reads were mapped to the *Mus musculus* mm10 reference genome using hisat2 2.1.0 with default parameters [64]. Multimapping reads were filtered using SAMtools 1.9 [65,66]. Uniquely aligned reads were then mapped to gene features using subread featureCounts v1.6.2 as a second strand library with parameters [67]. "-t gene -g gene_id -f -p -B -P -C." Differential expression between groups of samples was tested using R version 3.5.1 (2018-07-02) with DESeq2 1.20.0 [68]. Transcript quantification was performed with salmon 0.11.3 with parameters [69] "—gcBias—libType A—seqBias—threads 1." piRNA annotations were derived from the Zamore lab [5,70]. Transposon-mapping reads were aligned to repBase annotated regions [71], upbuilt from mm9 to mm10, using the software pipeline piPipes and bowtie2 2.2.5 [72].

### Small RNA-seq data analysis

After removing adaptors, rRNA and tRNA sequences were filtered. The remaining reads with sizes from 26 to 31 nt were mapped to the UCSC mm 10 assembly using hisat2 2.1.0 [64] and only uniquely mapped reads were used for further analysis. Through miRNA counts (miRbase) normalization piRNA abundance was obtained.

### Quantitative real-time RT-PCR (qRT-PCR)

Total RNA was isolated from mouse tissues using a miRNAeasy Mini Kit (Qiagen) and cDNA was synthesized with a RevertAid Premium First Strand cDNA Synthesis Kit (Thermo Fisher Scientific). Quantitative RT-PCR was performed using iTaq Universal SYBR Green Supermix (Bio-Rad) and QuantStudio 6 Flex Real-Time PCR System (Thermo Fisher Scientific). The relative abundance of each transcript was calculated by the 2^−ΔΔ*Ct*^ normalized to endogenous *β-actin* expression [73] and primer sequences are provided in S8 Table.

### Tandem mass tag (TMT) mass spectrometry

Mass spectrometry was performed at the Harvard FAS Division of Science Mass Spectrometry and Proteomics Resource Laboratory according to their posted protocols (https://proteomics.fas.harvard.edu/).

### Quantification and statistical analysis

All statistical analyses were performed using Graph Pad Prism 8 software. Comparisons between two experimental groups were made by the Mann-Whitney-Wilcoxon two-sided test and comparisons among three were made by one-way analysis of variance (ANOVA). No statistical methods were used to predetermine sample size, experiments were not randomized, and investigators were not blinded to allocation during experiments and outcome assessment, unless stated otherwise. Differences were considered significant at a level of *P* < 0.05.

## Supporting information

S1 Fig Generation of *pi18^Δ/Δ^* mice. (A) Schematic diagram of *pi18* piRNA coding locus deleted using CRISPR/Cas9. Scissors, sgRNAs target sites used to guide the Cas9-catalyzed promoter; red boxes, deletion (B) Genotyping of mutant founders by PCR. (C) Genomic sequences of *pi18* piRNA promoter region in *pi18^Δ/Δ^*. Dashes, genomic sequences deleted by CRISPR; blue NGG is protospacer adjacent motif (PAM); underlined, sgRNA.
(TIF)

S2 Fig Histological defects and increased apoptosis in *pi18^Δ/Δ^* mice. (A) TUNEL staining (top) and quantification (bottom) of TUNEL-positive tubules per cross-section from 8 wk/old mice (*n* = 3). Black arrowheads, apoptotic cells. Scale bar, 50 μm. The box indicates mean ± interquartile range, the whiskers indicate the highest/lowest values and middles are median values. **P < 0.01 (B) Testicular sections from 8 wk/old mice were stained with periodic acid-Schiff (PAS) and hematoxylin (H) to determine stages of seminiferous epithelium cycles (*n* = 3). Pl, preleptotene; L, leptotene; Z, zygotene; PS, pachytene spermatocytes; D, diplotene; RS, round spermatids; ES, elongating spermatids. Scale bar, 50 μm. Stage of seminiferous epithelium cycles was determined by morphology of spermatocytes and round spermatids. (C) Representative light microscopic images of testicular sections from 8 wk/old mice (*n* = 3 for each genotype). RS, round spermatid; ES, elongating spermatid; arrow heads, vacuolation. Scale bar, 50 μm; inset scale bar, 5 μm. The insets are enlargements of the dashed box regions. (D) PAS&H staining of epididymides from 12 wk/old mice (*n* = 2). Black arrowheads, sloughing germ cells. The insets are enlargements of the dashed box regions. Scale bar, 50 μm; inset scale bar, 5 μm.
(TIF)

S3 Fig Acrosome defects in *pi18^Δ/Δ^* spermatids. (A) Representative light microscopic images of PAS&H stained testicular sections to evaluate acrosome biogenesis: Golgi, cap, and acrosome phases from 8 wk/old control and mutant mice (*n* = 3). Pl, preleptotene; PS, pachytene spermatocytes; RS, round spermatids; ES, elongating spermatids; arrow heads, acrosome. The insets are enlargements of the dashed box regions. Scale bar, 50 μm; inset scale bar, 5 μm. S2-8, step 2–8 round spermatids; S10, step 10 elongating or elongated spermatids. Stage of seminiferous epithelium cycles was determined by morphology of spermatocytes and round spermatids. (B) Same as (A), but stained with PNA (acrosome, green) and Hoechst 33342 (DNA, blue).
(TIF)

S4 Fig Acrosome defects in *pi18^Δ/Δ^* spermatid development. (A) Representative TEM images of Golgi phase spermatids from 12 wk/old control and mutant mice (*n* = 3 for each genotype). N, nucleus; G, Golgi apparatus; C, *cis*-Golgi network; T, *trans*-Golgi network; PV, proacrosomal vesicle; CB, chromatoid body; AC, acrosome; Ag, acrosomal granule; Av, acrosomal vesicle; Apx, acroplaxome; M, manchette. Scale bar, 0.5 μm. ImageJ was used to quantify the density of ag (black area) and av (yellow dashed area) (top) and measure the nuclear perimeter (yellow and purple area) and length of Apx (yellow line) (bottom) in Fig 4 of manuscript. (B) Same as (A), but of cap phase spermatids. (C) Same as (B), but of acrosome phase spermatids.
(TIF)

S5 Fig Altered abundance of transcripts in *pi18^Δ/Δ^* mice. (A) MA-plots of transcripts in *pi18*^*+/+*^ compared to *pi18****^Δ/Δ^*** (left), *pi18****^+/Δ^*** mice (middle), and *pi18^+/Δ^* compared to *pi18^Δ/Δ^* (right) determined by RNA-seq. The y-axis is the log_2_ fold change in expression and the x-axis is averaged expression in both genotypes. Each point represents a transcript. Transcripts with *P* adjusted values < 0.1 are colored in red. (B) Using small RNA-seq, piRNAs were compared between *pi18^+/Δ^* and *pi18^Δ/Δ^* mice. Green dot, *pi18* piRNAs. (C) Gene ontology of differentially expressed genes after comparison of *pi18^+/+^* to *pi18^Δ/Δ^* mice. (D) Representative confocal microscopic images of meiotic chromosome spreads of *pi18^Δ/Δ^* pachytene (early, mid, late) spermatocytes from 4 wk/old mice. γH2AX, DNA damage marker (red) and SYCP3, synaptonemal complex protein 3 (green) on left. SYCP1, synaptonemal complex protein 1 (red) and SYCP3 on right. Arrowheads, sex chromosome; stage of pachytene spermatocytes was determined by desynapsis of sex chromosomes and SYCP3 staining.
(TIF)

S6 Fig Aberrant GOLGA2 expression in *pi18^Δ/Δ^* testes. (A) Venn diagrams depicting the overlap of comparative proteomes and transcriptomes of *pi18^+/Δ^* versus *pi18^Δ/Δ^* mice. (B) Representative confocal microscopic images of round and elongating spermatid heads from control and mutant mice (*n* = 3 for each genotype). Testicular germ cells were stained for GOLGA2 (red), PNA (acrosome, green) and Hoechst 33342 (nuclear DNA, blue), DIC, differential interference contrast. White arrowheads, GOLGA2 in depleted cytoplasm. Scale bar, 5 μm.
(TIF)

S1 Table DEG WT vs. HT. (XLSX)

S2 Table DEG WT vs. KO. (XLSX)

S3 Table DEG HT vs. KO. (XLSX)

S4 Table Transposon mapping. (XLSX)

S5 Table Functional enrichment. (XLSX)

S6 Table TMT Testis. (XLSX)

S7 Table TMT Sperm. (XLSX)

S8 Table Oligonucleotides. (DOCX)

S9 Table Antibodies. (DOCX)
