# Supplementary figures and images for: Sperm acrosome overgrowth and infertility in mice lacking chromosome 18 pachytene piRNA
Source: PLoS Genet. 2021 Apr 8;17(4):e1009485. doi: 10.1371/journal.pgen.1009485 (PMC8057611; doi:10.1371/journal.pgen.1009485)

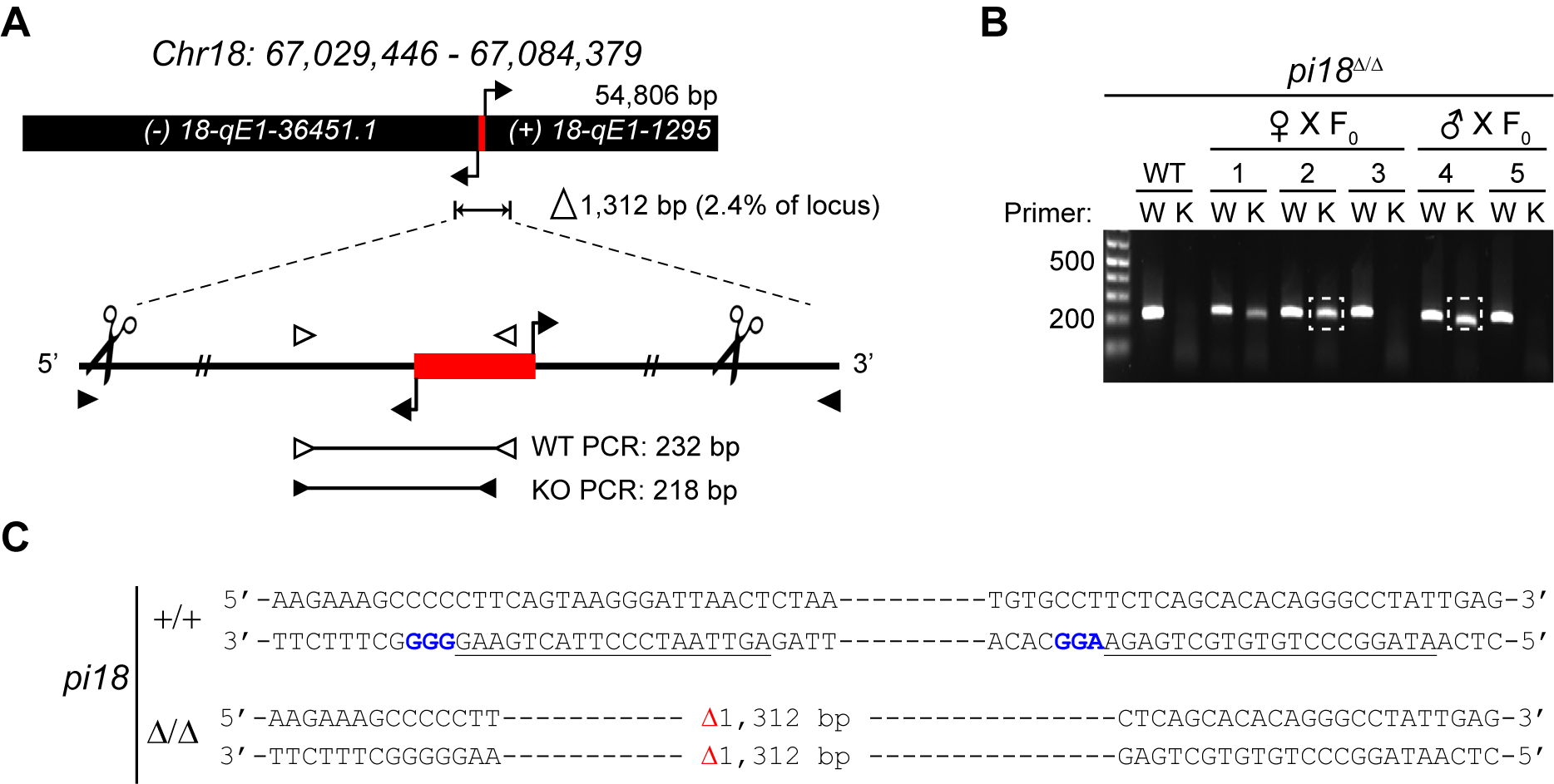

Supplement: S1 Fig — (A) Schematic diagram of pi18 piRNA coding locus deleted using CRISPR/Cas9. Scissors, sgRNAs target sites used to guide the Cas9-catalyzed promoter; red boxes, deletion (B) Genotyping of mutant founders by PCR. (C) Genomic sequences of pi18 piRNA promoter region in pi18Δ/Δ. Dashes, genomic sequences deleted by CRISPR; blue NGG is protospacer adjacent motif (PAM); underlined, sgRNA. (TIF) [file pgen.1009485.s001.tif]

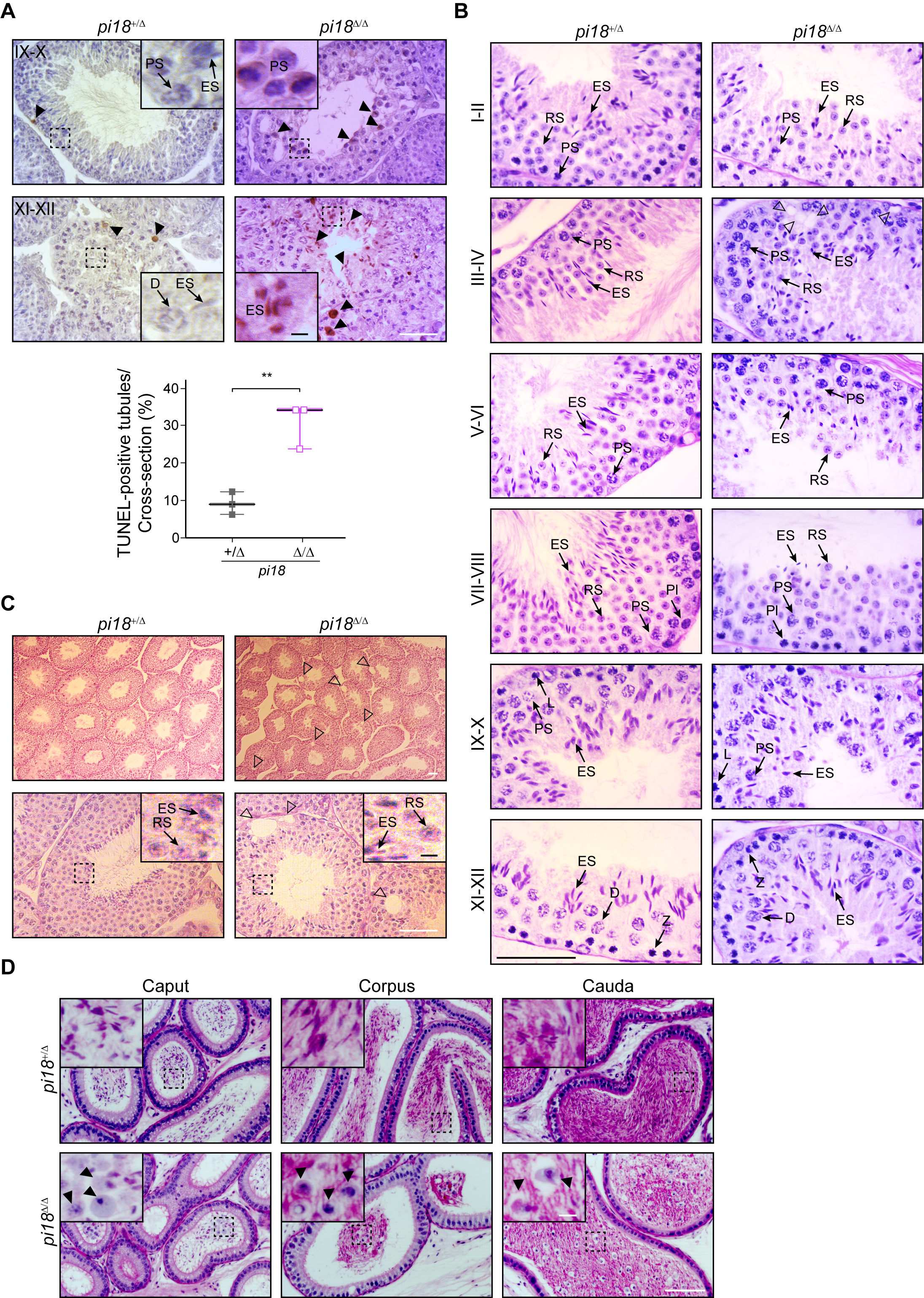

Supplement: S2 Fig — (A) TUNEL staining (top) and quantification (bottom) of TUNEL-positive tubules per cross-section from 8 wk/old mice (n = 3). Black arrowheads, apoptotic cells. Scale bar, 50 μm. The box indicates mean ± interquartile range, the whiskers indicate the highest/lowest values and middles are median values. **P < 0.01 (B) Testicular sections from 8 wk/old mice were stained with periodic acid-Schiff (PAS) and hematoxylin (H) to determine stages of seminiferous epithelium cycles (n = 3). Pl, preleptotene; L, leptotene; Z, zygotene; PS, pachytene spermatocytes; D, diplotene; RS, round spermatids; ES, elongating spermatids. Scale bar, 50 μm. Stage of seminiferous epithelium cycles was determined by morphology of spermatocytes and round spermatids. (C) Representative light microscopic images of testicular sections from 8 wk/old mice (n = 3 for each genotype). RS, round spermatid; ES, elongating spermatid; arrow heads, vacuolation. Scale bar, 50 μm; inset scale bar, 5 μm. The insets are enlargements of the dashed box regions. (D) PAS&H staining of epididymides from 12 wk/old mice (n = 2). Black arrowheads, sloughing germ cells. The insets are enlargements of the dashed box regions. Scale bar, 50 μm; inset scale bar, 5 μm. (TIF) [file pgen.1009485.s002.tif]

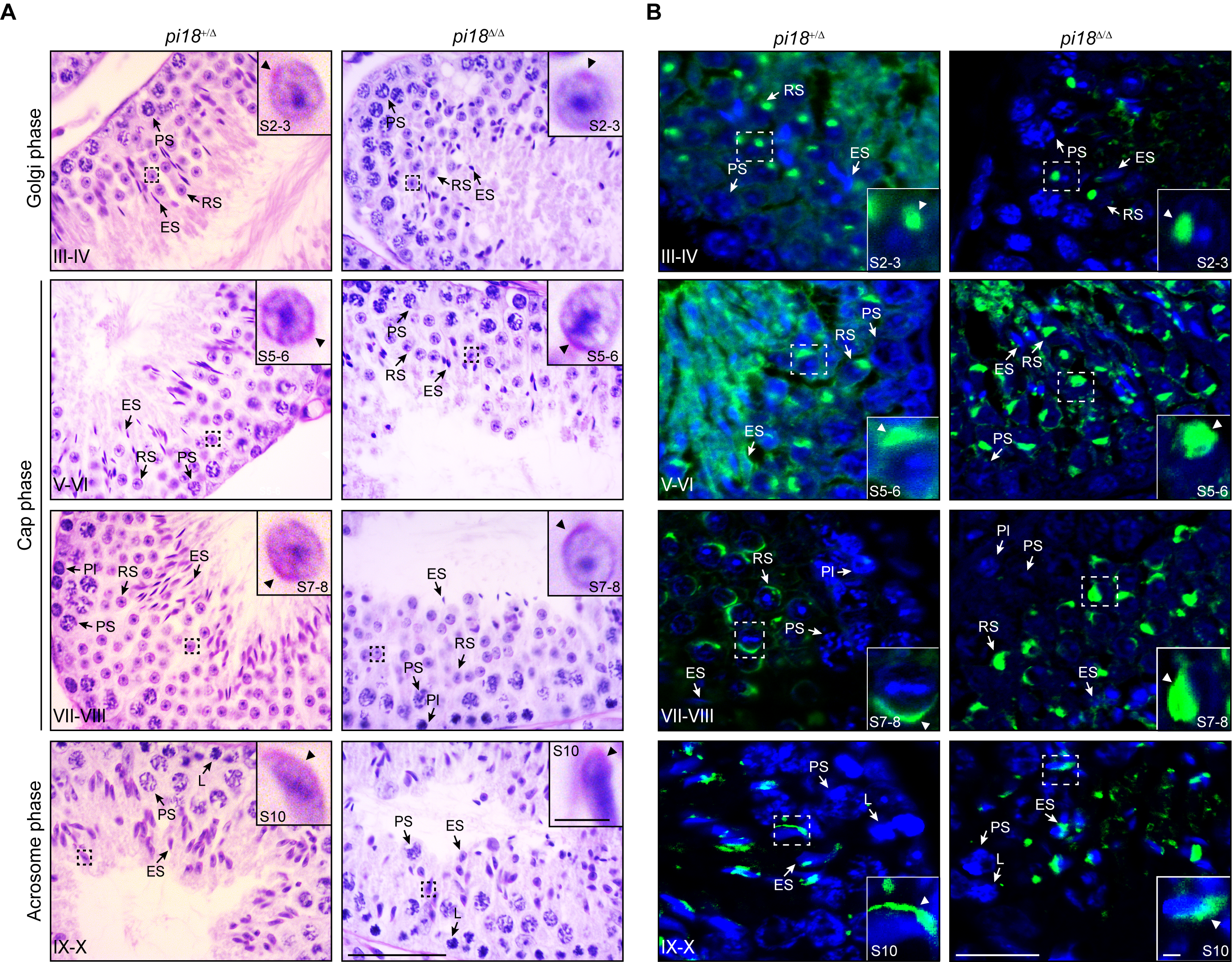

Supplement: S3 Fig — (A) Representative light microscopic images of PAS&H stained testicular sections to evaluate acrosome biogenesis: Golgi, cap, and acrosome phases from 8 wk/old control and mutant mice (n = 3). Pl, preleptotene; PS, pachytene spermatocytes; RS, round spermatids; ES, elongating spermatids; arrow heads, acrosome. The insets are enlargements of the dashed box regions. Scale bar, 50 μm; inset scale bar, 5 μm. S2-8, step 2–8 round spermatids; S10, step 10 elongating or elongated spermatids. Stage of seminiferous epithelium cycles was determined by morphology of spermatocytes and round spermatids. (B) Same as (A), but stained with PNA (acrosome, green) and Hoechst 33342 (DNA, blue). (TIF) [file pgen.1009485.s003.tif]

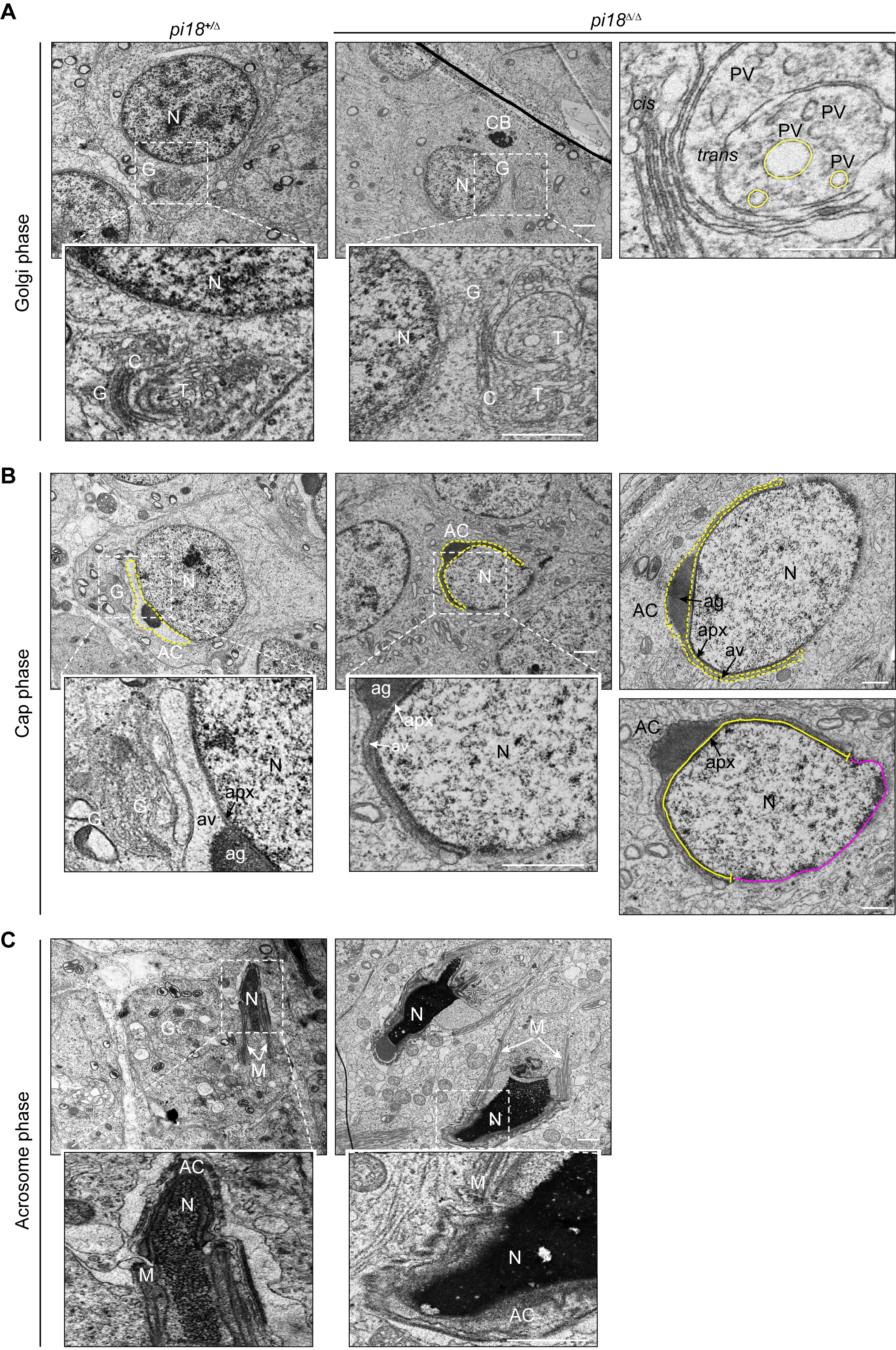

Supplement: S4 Fig — (A) Representative TEM images of Golgi phase spermatids from 12 wk/old control and mutant mice (n = 3 for each genotype). N, nucleus; G, Golgi apparatus; C, cis-Golgi network; T, trans-Golgi network; PV, proacrosomal vesicle; CB, chromatoid body; AC, acrosome; Ag, acrosomal granule; Av, acrosomal vesicle; Apx, acroplaxome; M, manchette. Scale bar, 0.5 μm. ImageJ was used to quantify the density of ag (black area) and av (yellow dashed area) (top) and measure the nuclear perimeter (yellow and purple area) and length of Apx (yellow line) (bottom) in Fig 4 of manuscript. (B) Same as (A), but of cap phase spermatids. (C) Same as (B), but of acrosome phase spermatids. (TIF) [file pgen.1009485.s004.tif]

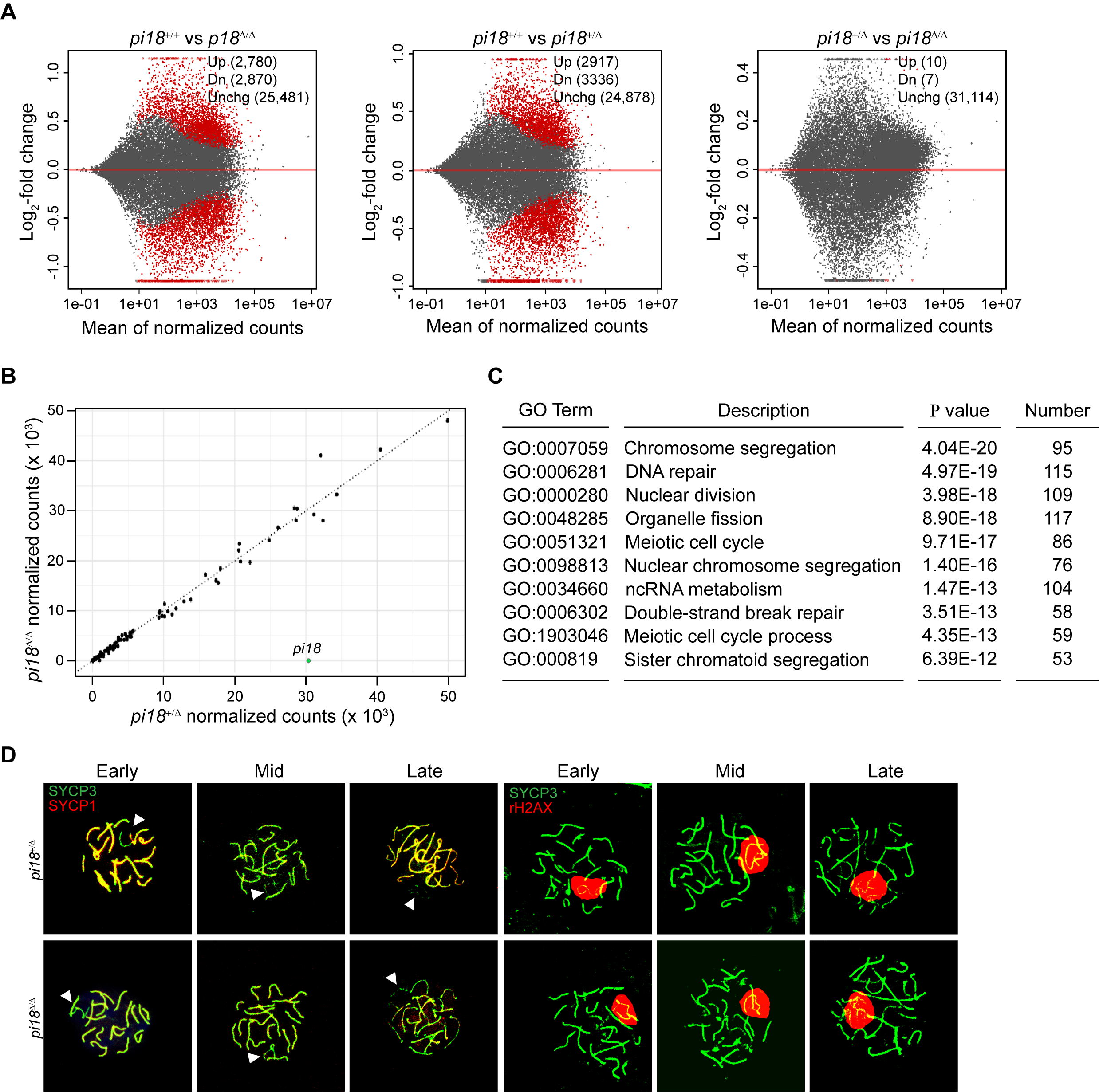

Supplement: S5 Fig — (A) MA-plots of transcripts in pi18+/+ compared to pi18Δ/Δ (left), pi18+/Δ mice (middle), and pi18+/Δ compared to pi18Δ/Δ (right) determined by RNA-seq. The y-axis is the log2 fold change in expression and the x-axis is averaged expression in both genotypes. Each point represents a transcript. Transcripts with P adjusted values < 0.1 are colored in red. (B) Using small RNA-seq, piRNAs were compared between pi18+/Δ and pi18Δ/Δ mice. Green dot, pi18 piRNAs. (C) Gene ontology of differentially expressed genes after comparison of pi18+/+ to pi18Δ/Δ mice. (D) Representative confocal microscopic images of meiotic chromosome spreads of pi18Δ/Δ pachytene (early, mid, late) spermatocytes from 4 wk/old mice. γH2AX, DNA damage marker (red) and SYCP3, synaptonemal complex protein 3 (green) on left. SYCP1, synaptonemal complex protein 1 (red) and SYCP3 on right. Arrowheads, sex chromosome; stage of pachytene spermatocytes was determined by desynapsis of sex chromosomes and SYCP3 staining. (TIF) [file pgen.1009485.s005.tif]

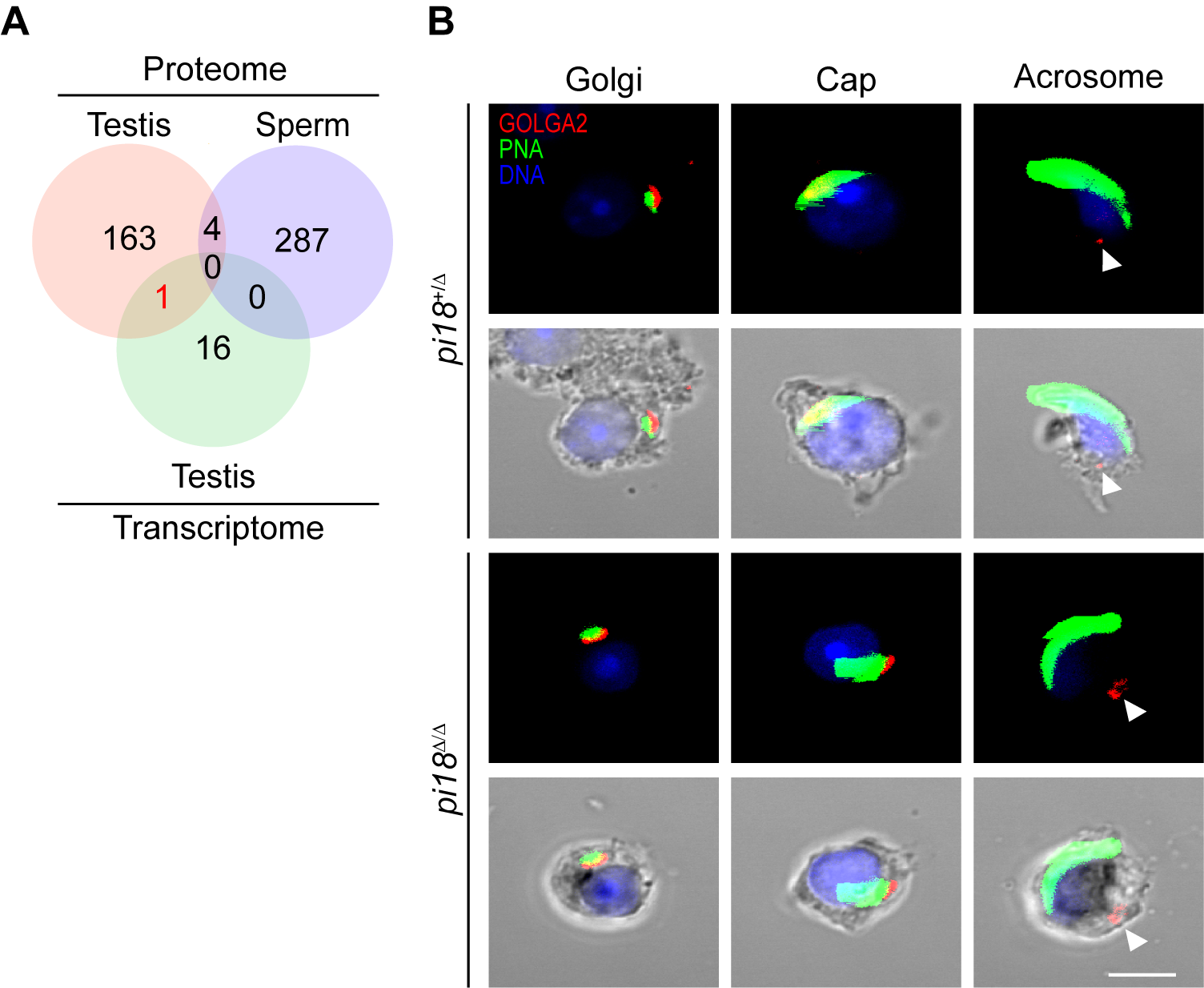

Supplement: S6 Fig — (A) Venn diagrams depicting the overlap of comparative proteomes and transcriptomes of pi18+/Δ versus pi18Δ/Δ mice. (B) Representative confocal microscopic images of round and elongating spermatid heads from control and mutant mice (n = 3 for each genotype). Testicular germ cells were stained for GOLGA2 (red), PNA (acrosome, green) and Hoechst 33342 (nuclear DNA, blue), DIC, differential interference contrast. White arrowheads, GOLGA2 in depleted cytoplasm. Scale bar, 5 μm. (TIF) [file pgen.1009485.s006.tif]
