# Supplementary material for: Sperm acrosome overgrowth and infertility in mice lacking chromosome 18 pachytene piRNA
Source: PLoS Genet. 2021 Apr 8;17(4):e1009485. doi: 10.1371/journal.pgen.1009485 (PMC8057611; doi:10.1371/journal.pgen.1009485)
Supplement: S8 Table — (DOCX) [file pgen.1009485.s014.docx]

**S8 Table. Oligonucleotides**

| **#** | **Name** | **Sequence (5'-3')** | **Source** | **Description** |
| --- | --- | --- | --- | --- |
| 1  2 | pi18_WT_F  pi18_WT_R | TTCCAGCTGTTCACAAGACG  CAGTTGATGGGCAGCAGTTA | IDT | Genotyping PCR |
| 3  4 | pi18_KO_F  pi18_KO_R | CACTTCTTCTGCCGAATCCT  ATGAGCTGAAGCAGGGATGT | IDT | Genotyping PCR |
| 5  6 | Golga2_F  Golga2_R | AACGGCAGGTACAAGAGCTGGA  CTCTCCAGTTCCTTGTGCAGCT | IDT | qRT-PCR |
| 7  8 | Vps54_F  Vps54_R | CAAGGAGCAGACATCAGCGTGT  GCATTACACAGTAACTCCTGGATG | IDT | qRT-PCR |
| 9  10 | Cdc42_F  Cdc42_R | GATTGGTGGAGAGCCATACACTC  TGAGGATGGAGAGACCACTGAG | IDT | qRT-PCR |
| 11  12 | Hsp90b1_F  Hsp90b1_R | GTTTCCCGTGAGACTCTTCAGC  ATTCGTGCCGAACTCCTTCCAG | IDT | qRT-PCR |
| 13  14 | Gopc_F  Gopc_R | GCGTCAGAAGATGACCAGCCTT  GATCGCAGATCCACCAACTGTG | IDT | qRT-PCR |
