# Supplementary material for: Sperm acrosome overgrowth and infertility in mice lacking chromosome 18 pachytene piRNA
Source: PLoS Genet. 2021 Apr 8;17(4):e1009485. doi: 10.1371/journal.pgen.1009485 (PMC8057611; doi:10.1371/journal.pgen.1009485)
Supplement: S9 Table — (DOCX) [file pgen.1009485.s015.docx]

**S9 Table. Antibodies**

| **Antibody Name** | | **Source** | **Cat #** |
| --- | --- | --- | --- |
|  |  |  |  |
|  | Rabbit polyclonal anti-β-actin | Cell Signaling Technology | 4970 |
|  | Rabbit monoclonal anti-LINE-1 ORF1p | Abcam | Ab216324 |
| **GOLGA2/GM130** | Rabbit monoclonal anti-GM130 | Abcam | ab52649 |
|  | Mouse anti-GM130 | BD Transduction Laboratories | 610823 |
|  | PNA-FITC | Amsbio | AMS.JOM-J5S1 |
|  | Rabbit polyclonal anti-SYCP-1 | Novus Biologicals | NB300-229 |
|  | Mouse monoclonal anti-SYCP-3 | Santa Cruz | SC-74569 |
|  | Mouse monoclonal anti-ɤH2AX | Millipore Sigma | 05-636 |
|  | Goat anti-rabbit IgG (H+L) cross-absorbed secondary antibody, Alexa Fluor 594 | ThermoFisher Scientific | A-11012 |
|  | Goat anti-mouse IgG (H+L) cross-absorbed secondary antibody, Alexa Fluor 568 | ThermoFisher Scientific | A-11004 |
|  | Goat anti-mouse IgG (H+L) cross-absorbed secondary antibody, Alexa Fluor 488 | ThermoFisher Scientific | A-11001 |
|  | Goat anti-rabbit IgG (H+L), HRP conjugate | Promega | W4011 |
|  | Goat anti-mouse IgG (H+L), HRP conjugate | Promega | W4021 |
